# Transcriptomic divergences of larval labial salivary glands facilitate host-plant range oscillations between specialist and generalist *Helicoverpa* species

**DOI:** 10.1186/s12864-026-12774-z

**Published:** 2026-04-02

**Authors:** Xiongya Wang, Lihong Yang, Zhongyuan Deng, Luciano M Matzkin, Xianchun Li

**Affiliations:** 1 Zhengzhou Research Base, National Key Laboratory of Cotton Bio-breeding and Integrated Utilization, School of Agriculture and Biomanufacturing, Zhengzhou University, Zhengzhou, 450052 China; 2 Institute of Biology Co., Ltd, Henan Academy of Sciences, Zhengzhou, 450008 Henan China; 3 Department of Entomology, University of Arizona, Tucson, AZ 85721 USA

**Keywords:** Host modification, Host range oscillation, Plant defense, Pseudogenization, Secretome size, Transposable elements

## Abstract

Host plant range of insect herbivores may evolve towards generalization and specialization. However, how generalist herbivores achieve polyphagy and what facilitate host-plant range oscillation remains elusive. Our recent finding of a negative correlation between host-plant range and genome size in the *Helicoverpa* lineage predicts that the generalist *Helicoverpa armigera*, rather than its ancestral specialist sibling *Helicoverpa assulta*, should have a larger secretome size to modify host defenses, nutrients and/or structure, and should purge a significant amount of transposable elements (TEs) to minimize pseudogenization of genes associated with host utilization. To test these two predictions, we compared the sizes and compositions of the larval labial salivary gland transcriptomes of these two species, as well as the numbers of pseudogenes in four host defense detoxification gene families in their genomes. Indeed, the labial salivary gland transcriptome of *H. armigera* was significantly greater than that of *H. assulta* at both the contig and gene levels, especially for the Gene Ontology (GO) terms and KEGG pathways associated the main functions of the larval salivary gland, such as protein synthesis and secretion, herbivore offence, digestion, detoxification. By contrast, *H. assulta* had more contigs inserted with TEs, more transcripts involved in transposition of TEs, and more pseudogenized host defense detoxification genes. These results not only match the predictions but also demonstrate that oscillation of host plant range evolution in *Helicoverpa* species is at least in part facilitated by TE-mediate pseudogenization (shutdown) and reactivation (on) of host use and secretion-related genes.

**Supplementary Information:**

The online version contains supplementary material available at 10.1186/s12864-026-12774-z.

## Lay summary

Diet breadth of plant-eating insects oscillates between generalization and specialization. How generalist plant feeders achieve wide diet breadth and what facilitate oscillation of diet breadth remains elusive. In this study, we selected two sibling species of the cotton bollworm (CBW) lineage to address the above general questions. Our recent finding of a negative correlation between the diet breadth and the size of the repertoire of genetic materials (i.e. DNA, which encodes basic genetic units called genes) in the CBW lineage predicts that generalist CBW species should have a larger set of secreted proteins than their specialist siblings to modify plant structures, nutrients and/or defenses (Zhang et al., 2019) [1]. Here we show that the generalist CBW expressed 1.88, 1.50, 2.52, 2.33 and 1.85 times more species-specific genes, essential genes, environmental response genes, gene readouts and putative secreted protein genes in the larval secretion tissue than did its ancestral specialist sibling oriental tobacco budworm (TBW). The CBW / TBW species-specific gene readout ratio jumped to 4.4–30.0 for genes involved in insect offense, digestion and/or detoxification, and 4.8–70.8 for those associated with other major functions (protein synthesis and secretion) of the larval secretion tissue. CBW also tended to have more alternative versions (the lay term for different alleles of a gene) and higher expressions for these genes than did TBW. By contrast, TBW expressed more readouts involved in transposition of jumping genes, i.e., transposable elements (TEs). These results support the prediction and show that diet breadth oscillation in this lineage is accompanied by TE-mediated off and reactivation of plant use and secretion related genes.

## Teaser text

Insect herbivores, particularly those with a broad host-plant range, cause serious damages to agriculture. The host-plant range of insect herbivores may oscillate between generalization and specialization. How generalist herbivores achieve polyphagy and what facilitate oscillation of host-plant range evolution remains largely unknown. This study chose the polyphagous *Helicoverpa armigera* and its ancestral specialist sibling *Helicoverpa assulta* to demystify host-range oscillation, a common ecological, evolutionary, and genomic/transcriptomic event that occurs frequently across a broad range of taxa, including herbivores, predators, parasitoids, and pathogens of plants and animals. The results reveal that oscillation of host plant range in the *Helicoverpa* lineage is accompanied by transposon-mediate pseudogenization (off) and reactivation (on) of host use and secretion related genes. This pioneer study represents to our knowledge the first report of the mechanisms that facilitate oscillation of host-plant range evolution in herbivorous insects.

## Introduction

Host range is known to oscillate between host generalization and host specialization [2–7], which represent two opposite directions of host range evolution [8]. Relative to host specialists, generalists have to cope with an enormous diversity of host nutrients and defenses. This may be achieved by adapting to divergent host environments via evolving additional host use related genes involved in digestion and detoxification, or by modifying distinct host environments to one or a few standard host nutrient/defense environments to which generalists are specialized via secretion of effectors, toxins and host-cell interaction proteins [9–14]. Generalists that rely on the former—host adaptation—to expand host range are expected to have larger genome size and more expressed genes involved in digestion and detoxification. By contrast, those that depend on host modification/standardization via secretion to broaden host range should have a larger size of secretome (the complete collection of the expressed genes and alleles coding for secreted proteins in the secretary tissue larval labial salivary gland) [12].

Utilization of host plants by herbivorous insects involves antennae, labial salivary glands, mandibular glands, midgut, and fat body. Using the host adaptation and modification definitions described above, it is expected that labial salivary glands and, to a lesser extent, mandibular glands, would be the two major tissues involved in host plant modification [1] as they express and secrete effector gene products (e.g., glucose oxidase, ATP hydrolyzing enzymes, calcium-binding proteins, ferritin, Bt56, secreted host-responsive protein in Tetranychidae, MpC002) into saliva or oral secretions to manipulate/modify host plant nutrients, structure and defense [15–25]. The other three tissues (i.e., antennae, midgut, and fat body) function largely as host adaptation tissues [1]. Specifically, antennae (to a less extent, maxilla and ovipositors as well) express host odor-perceiving odorant-binding proteins (OBPs), odorant receptor (ORs), ionotropic receptor (IRs) and chemosensory proteins (CSPs) to locate suitable host plants and to avoid unsuitable hosts [26–28]. Midgut produces digestion enzymes such as amylases, glycosidases, proteinases, and lipases to digest plant tissues/nutrients and plant defense proteins [29–31]. Fat body and midgut express detoxification enzymes such as cytochrome P450 monooxygenases (P450s), glutathione-S-transferases (GSTs), carboxylesterase (CCEs), and UDP-glycosyltransferases (UGTs) to detoxify toxic plant defensive allelochemicals [8, 15, 32–35]. Midgut also acts as a minor host modification tissue to contribute part of its luminal contents into saliva [36, 37]. If host modification is the main route to host generalism, a generalist should have a larger secretome size than its specialist counterpart. If this is not the major mechanism of generalization, then a generalist would have a relatively larger repertoire of expressed genes relating to digestion, detoxification and/or olfaction.

*Helicoverpa assulta* and its sympatric descendant *Helicoverpa armigera* are a pair of closely related hybridizable species that differ mainly in diet breadth [38–42]. *H. assulta* is a Solanaceae specialist feeding only on tobacco, pepper, tomato, and eggplants, whereas the more derived *H. armigera* is a generalist capable of feeding on hundreds of plant species that belong to about 30 different plant families, including Malvaceae, Leguminosae, Gramineae and Solanaceae [38, 41, 43–47]. The fact that genome size inversely correlates with host plant range in *Helicoverpa* species [1] suggests that host modification is the main route to expansion of host plant range in this lineage, and thus *H. armigera* should have a larger size of secretome than *H. assulta*. To test these predictions, we compared the size and compositions of the labial salivary gland transcriptome of the two species and their numbers of pseudogenes in the P450, CCE, UGT and GST gene families. Unlike the transcriptomes of the sex pheromone gland, sex pheromone-ovipositor complex, and antennae which exhibit little differences between *H. armigera* and *H. assulta* [48–50], significantly more transcripts and genes involved in the GO terms and KEGG pathways associated the main functions of the larval salivary gland, such as protein synthesis and secretion, herbivore offence, digestion, detoxification were detected in the labial salivary gland transcriptome of *H. armigera*, relative to that of *H. assulta*. By contrast, the main Gene Ontology (GO) terms with more transcripts in *H. assulta* than in *H. armigera* were involved in transposition of transposable elements (TEs), rather than in host use or other essential biological processes. Moreover, *H. assulta* also had significantly more contigs inserted with TEs and significantly more pseudogenized members in the four detoxification gene families. These results not only support the suggestion that *H. armigera* relies more on host modification to expand its host plant range, but also imply that oscillation of host plant range in the *Helicoverpa* lineage is facilitated, at least partially, by TE-mediate pseudogenization (a key evolutionary process where transposable elements insert into existing genes, causing them to become non-functional pseudogenes) (shutdown) and reactivation (on) of host use and secretion related genes.

## Materials and methods

### Insects

The laboratory strains of *H. armigera* and *H. assulta* were established with adults of each species developed from a total of 2100 larvae of the two species (indiscernible at larval stage) collected on tobacco plants from Xuchang (Henan, China) in June 2015 [51]. The two strains had been kept on wheat germ-containing artificial diet [52] (larvae) or 8% sugar water with 2% honey at 27 ± 1 °C, 70 ± 10% (for adults) or 40 ± 10% (for larvae) RH and a photoperiod of 16 L:8 D since then.

### Dissection of labial salivary glands and RNA extraction

A total of 99 and 89 pairs of labial salivary glands were individually dissected out from 48 h-old 6th instar larvae of *H. armigera* and *H. assulta*, respectively, washed three times to remove potential hemocytes with pre-cold sterilized 1× Phosphate Buffered Saline (1× PBS) solution, flash-frozen in liquid nitrogen, and stored at -80 °C prior to RNA extraction. Total RNAs were extracted from the two labial salivary gland samples as described in Li et al. [53]. Potential genomic DNA contaminants in each total RNA sample were removed by digestion at 37 °C for 40 min in 200 µL reaction containing 20 µg RNA sample, 10 µL RNase inhibitor (20 U/ µL, Thermo, USA) and 4 µL RQ1 RNase-free DNase I (Promega, USA). Each reaction mixture was extracted with phenol/chloroform to obtain DNA-free total RNA. The purity and integrity of the resultant DNA-free total RNA samples were evaluated by NanoDrop 2000 (NanoDrop, USA) and RNA Nano 6000 Assay Kit of the Bioanalyzer 2100 system (Agilent Technologies, CA, USA), respectively, and stored at -80 °C for subsequent RNA sequencing (RNA-seq). Additionally, we extracted and stored three total RNA samples of 15 pairs of labial salivary glands each from 48 h-old 6th instar larvae of *H. armigera* or *H. assulta* for subsequent quantitative reverse transcription PCR (RT-qPCR) verification of 6 differentially expressed orthologous genes (Sect.  2.8).

### Illumina Library preparation and sequencing

Two index-coded sequencing libraries were generated from 1 µg of *H. armigera* and *H. assulta* labial salivary glands RNA samples, respectively, using TruSeq RNA Sample Preparation Kit (Illumina, San Diego, CA, USA) at Sinobiocore (Beijing, China). Briefly, mRNA was purified from each total RNA sample using poly-T oligo-attached magnetic beads and fragmented into small pieces using divalent cations under elevated temperature in an Illumina proprietary fragmentation buffer. The cleaved mRNA fragments were reversed-transcribed into first strand cDNA using SuperScript II reverse transcriptase and random primers, followed by synthesis of second strand cDNA using DNA Polymerase I and RNase H. The cDNA fragments were end-blunted with DNA polymerase I, 3′-adenylated, and ligated with index-coded adapters. The adaptor-ligated cDNA fragments of preferentially 250–300 bp were size selected from each ligation reaction with AMPure XP system (Beckman Coulter, Beverly, MA, USA) and selectively enriched by 10 rounds of PCR amplification using Illumina PCR Primer Cocktail. The PCR products were purified with AMPure XP system to create the final cDNA library. The library quality and quantity were assessed using the Agilent high-sensitivity DNA assay on the Agilent Bioanalyzer 2100 system and using qPCR by StepOnePlusReal-Time PCR system (ABI, USA), respectively. The resultant index-coded *H. armigera* and *H. assulta* libraries were clustered on a cBot Cluster Generation System using TruSeq PE Cluster Kit v3-cBot-HS (Illumia, San Diego, USA) and then sequenced on an Illumina Hiseq 2000 platform to generate 150 bp paired-end reads.

### *De novo* transcriptome assembly, annotation and quantification of gene expression

Raw reads of each species were processed to get clean reads by trimming adaptor sequences and removing reads containing poly-N and low-quality reads ($\:\ge\:$ 50% bases with a Q value of ≤ 10). The resultant clean, high-quality reads of each species were *de novo* assembled as the initial contigs of *H. armigera* and *H. assulta* labial salivary gland transcriptomes, respectively, using Trinity (http://trinityrnaseq.sf.net). Transposable elements (TEs) present in the initial contigs of each species were identified by Repeat Modeler (http://www.repeatmasker.org/RepeatModeler.html) and BLAST search against the Repbase database (https://www.girinst.org/repbase/), respectively, yielding a merged TE database for each species. The initial contigs for each species were compared to the TE data base of the corresponding species using Repeatmasker [54] to mask TE sequences present in any contigs or filter TE-only contigs. This TE-masking step would prevent us from incorrectly identifying the contigs containing similar TEs as orthologues, paralogues or alleles in Sect.  2.5.

Burrows-Wheeler-Aligner (BWA) (0.7.5a-r405) was used to align clean reads to contigs. Cuffdiff (v2.2.1) was used to calculate the Reads Per Kilo base of exon model per Million mapped reads (RPKM) of contigs of each species based on the length of the contig and reads count mapped to each contig [55]. Contigs with a read count of less than 1 were filtered out and excluded from further analyses. To annotate the filtered contigs of both species, BLASTx and BLASTn searches were performed against the NCBI Nt and Nr databases and BLASTp against the KEGG Orthology (KO) and pathway databases (*e*-value < 10^–5^, bitscore > 60). The blast results were then imported into Blast2GO pipeline (http://www.blast2go.org/) for GO (Gene Ontology) annotations. The filtered contigs undergo pairwise comparison, and those exhibiting high sequence similarity (typically > 94%) and likely originating from the same gene are grouped into the same “cluster.” Each cluster is considered to represent a unigene.

### Identification of orthologues, paralogues, alleles and species-specifically expressed genes and enrichment analyses

Orthologues, paralogues, alleles and species-specifically expressed genes were identified by combining a reciprocal batch Blastn search between the TE-masked *H. armigera* contig database and the TE-masked *H. assulta* contig database and batch BlastN and BlastX searches of the NCBI nr/nt database using the TE-masked *H. armigera* or *H. assulta* contig database as the query. The *H. armigera* / *H. assulta* contig pairs that were the reciprocal best matches to each other and hit the same NCBI subject or its alleles with similar sequence identity and e-value constituted the majority of *H. armigera* / *H. assulta* orthologues. The unmatched *H. armigera* and *H. assulta* contigs that hit different NCBI subjects represented the transcripts or genes specifically expressed in *H. armigera* and *H. assulta* labial salivary gland, respectively. A small portion of the unmatched *H. armigera* and *H. assulta* contigs also hit the same NCBI subject or its alleles with similar sequence identity and e-value, and thus were considered as part of *H. armigera* / *H. assulta* orthologues whose corresponding *H. armigera* and *H. assulta* contigs were from different regions. The non-best matching contigs in the two species that hit the same NCBI subject or its paralogues /alleles with similar sequence identity and e-value were either paralogues or alleles. Based on our observation of sequence variations of multiple genes with *Helicoverpa* species, we arbitrarily designated *H. armigera* or *H. assulta* contigs that shared a nucleotide identity of < 98% and ≥ 98% as paralogues and alleles, respectively. The numbers of expressed orthologues and species-specific genes as well as the numbers of paralogues and alleles of each gene were counted and recorded.

Venn charts were drawn using Venny 2.1.0. (http://bioinfogp.cnb.csic.es/tools/venny/index.html) to exhibit the shared or species-specifically expressed genes between the two species. GO term and KEGG pathway enrichment analyses of species-specifically expressed transcripts or genes were performed online at https://www.omicshare.com/tools/Home/Soft/gogsea and https://www.omicshare.com/tools/Home/Soft/patwhwaygsea, respectively. GO terms or KEGG pathways with corrected p value less than 0.05 were considered to be significantly enriched among the species-specifically expressed transcripts or genes.

### Identification and comparison of essential vs. environment response genes

Genes that are indispensable for the survival, growth and reproduction of an organism are defined as essential genes, while genes involved in environmental adaption are defined as environment response genes [56]. A total of 208 environment response genes (see the environmental gene sheet in Dataset S1) selected from those encoding for digestive enzymes (e.g. proteases, carbohydrases and lipases), detoxification enzymes [e.g. cytochrome P450 monooxygenases (P450s), glutathione S-transferases (GSTs)] immune proteins (e.g. lectins, phenoloxidase, hemocytin) and saliva elicitors (e.g. glucose oxidase, beta-glucosidase, apyrase) were used to retrieve all the corresponding *H. armigera* and *H. assulta* contigs of the 208 genes. Likewise, a total of 606 *Drosophila melanogaster* essential genes (see the essential gene sheet in Dataset S1) downloaded from the Database of Essential Genes (http://www.essentialgene.org/; 339 genes) and the Online Gene Essentiality Database (https://v3.ogee.info/ #/home; 267 genes) were used to retrieve all the corresponding *H. armigera* and *H. assulta* contigs of the 606 genes. As described in Sect.  2.5, we further designated the retrieved essential and environment response gene contigs as shared orthologues, species-specific genes, paralogues and/or alleles, respectively.

### Bioinformatic identification and comparison of putative secreted proteins

Putative secreted proteins from *H. armigera* and *H. assulta* labial salivary glands were predicted as described in Boulain et al. [57]. All the transcribed contigs of the two sibling species were aligned by BLASTn to the *H. armigera* genome [58] to acquire the full-length open reading frame and thus translated into amino acid sequences of the corresponding contigs for prediction of secreted proteins. Signal peptides and non-classical secreted proteins were predicted by SignalP-5.0 Server http://www.cbs.dtu.dk/services/SignalP/ [59] and SecretomeP 2.0 Server (NN-score > 0.5) (http://www.cbs.dtu.dk/services/SecretomeP/) [60] respectively to identify all potential secreted proteins. The final expressed putative secreted proteins from *H. armigera* and *H. assulta* labial salivary glands were obtained by excluding those which contain membrane-inserted domains, such as transmembrane helices (predicted by TMHMM Server v. 2.0, http://www.cbs.dtu.dk/services/TMHMM/*)* [61, 62] or GPI anchors (predicted by GPI-SOM, http://gpi.unibe.ch/*)* [63]. As described in Sect.  2.5, we further designated the putative secreted proteins as shared orthologues, species-specific genes, paralogues and/or alleles, respectively.

### RT-qPCR verification of six orthologous genes

RNA-seq expression data of three known secreted orthologous protein genes including ecdysone oxidase (EO), yellow-d (YD) and serine protease inhibitor dipetalogastin precursor (SPI_D) [64] and three differentially expressed orthologous genes including cell wall protein IFF6 (CWP), lipase 3 (LP-3) and aldehyde dehydrogenase (AD) were verified by qRT-PCR using ribosomal protein L-13 (RPL-13), ribosomal protein L-32 (RPL-32) and β-Tubulin (β-TUB) as the internal reference genes. Each of the three replicate RNA samples of *H. armigera* or *H. assulta* was reverse-transcribed into cDNA samples at 42 °C for 1 h in a 20 µL reaction consisting of 1.5 µg RNA, 2 µL primer Mix (Tiangen, China), 4 µL dNTP, 1 µL M-MuLV reverse transcriptase (New England Biolab) and 1 µL RNase inhibitor (Thermo, USA). The qPCR reaction system of the six target genes and three reference genes contained 10 µL 2× SuperReal PreMix Plus (TIANGEN, China), 0.4 µL 50× ROX Reference Dye (TIANGEN, China), 2 µL gene-specific primers (1 µL each; Table S1), 1 µL cDNA template and 6.6 µL nuclease-free water. The qPCR reactions of the six target genes and three reference genes were performed in an ABI 7500 Real-Time PCR System (Applied Biosystems, Foster City, CA) with initial denaturation at 95 °C for 15 min, followed by 40 cycles of denaturation at 95 °C for 10 s, annealing at 55 °C for 30 s and extension at 72 °C for 32 s. Additional melting curve was set from 95 °C to 60 °C to ensure free of junk products. Amplification efficiency (E) of each gene (Table S1) was determined from the slope of the log template concentration (x-axis) - Ct value (y-axis) line using the formula E = 10 − 1/slope − 1 [65]. The normalized expression levels of the six target genes were calculated using the formula below:

Normalized expression level of each target gene:

$$\:=\frac{{\left(1+\mathrm{E}\:target\:\right)}^{-\mathrm{C}\mathrm{t}\:target}}{\sqrt[3]{{\left(1+\mathrm{E}\:RPL-32\right)}^{-\mathrm{C}\mathrm{t}\:RPL-32}*{\left(1+\mathrm{E}\:RPL-13\right)}^{-\mathrm{C}\mathrm{t}\:\:RPL-13}*{\left(1+\mathrm{E}\:\beta\:-tubulin\right)}^{-\mathrm{C}\mathrm{t}\:\beta\:-tubulin}}}$$

where E is amplification efficiency.

### Data analyses

The contents of TEs in the genomes of *H. armigera* and *H. assulta* were obtained from Zhang et al. (2023) [66] and Kim et al. (2024) [67], respectively. The numbers of pseudogenized and active members in the families of P450s, CCE, UST and GST of *H. armigera* and *H. assulta* were obtained from Zhang et al. (2024) [68] and Kim et al. (2024) [67] (see the annotation table “Hassulta_Detox genes_ASJ_19.04.24. xlsx” posted in the fgshare at https://doi.org/10.6084/m9. figshare.25650450), respectively. Cross-species differences in the TE content, the numbers of pseudogene in the P450, CCE, UGT and GST families and the number of contigs inserted with TEs were tested by Chi-square test (*P* < 0.05). Cross-species differences in the numbers of the species-specific total contigs, essential genes, environmental genes, and putative secreted protein genes were analyzed by Pearson Chi-square test (*P* < 0.05). Cross-species differences in the normalized expression levels of the 6 target genes as well as the RPKM values and the numbers of paralogues or alleles per shared or species-specific genes, essential genes, environmental genes, and putative secreted proteins genes were determined by t-tests (*P* < 0.05). All the statistical tests were done in SPSS software version 17.

## Results

### *H. armigera / H. assulta ratio* of the transcriptome sizes of their larval labial salivary glands

**Fig. 1:**
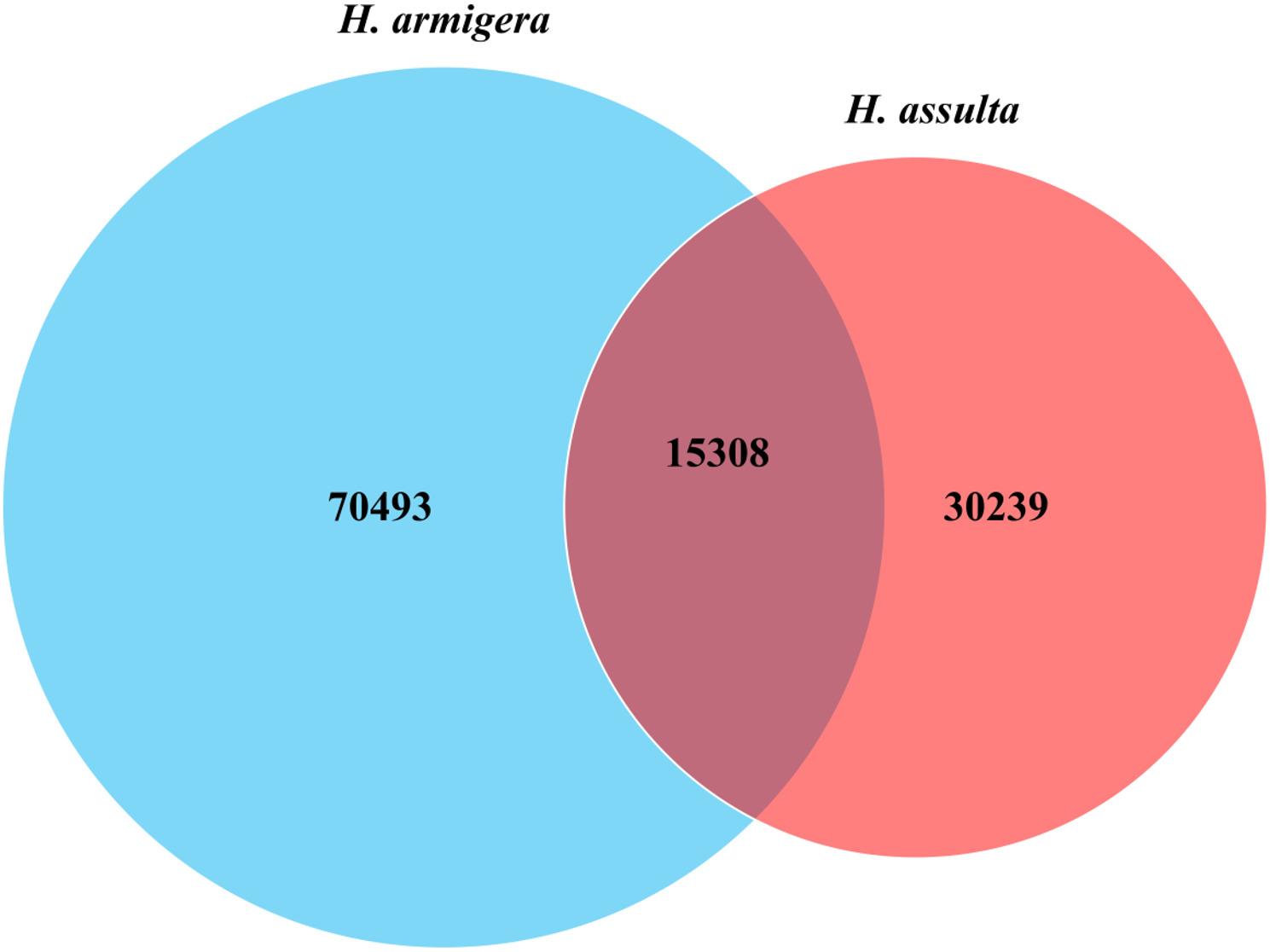
Venn diagram of the numbers of the shared and species-specific contigs expressed in labial salivary gland of *H. armigera* and *H. assulta.* The lists of the shared and species-specific contigs can be found in the corresponding sheets of Dataset S1

To test if the generalist *H. armigera* has a larger size of secretome (the complete collection of the expressed genes and alleles coding for secreted proteins in the secretary tissue larval labial salivary gland) than its specialist counterpart *H. assulta*, we compared the transcriptome sizes of their larval secretary tissue labial salivary glands. Illumina sequencing of the labial salivary gland RNA samples from 48 h-old 6th instar larvae of *H. armigera* and *H. assulta* yielded a total number of 38,149,826 and 36,178,424 clean reads (Table 1), respectively. While the numbers of clean reads and total length of the assembled bases obtained from the two sibling species were approximately the same (*H. armigera* / *H. assulta* read ratio = 38149826 / 36178424 = 1.06; *H. armigera* / *H. assulta* assembled base ratio = 51412837 / 46013942 = 1.12), the numbers of the assembled clean contigs and clustered unigenes (a set of contigs that appear to come from the same gene) from these clean reads for *H. armigera* were 1.88 (85801/45547) and 1.91 (69340/36345) times more than those for *H. assulta* (Table 1), respectively. Combination of reciprocal BLASTn between *H. armigera* contig database and *H. assulta* contig database as well as BLASTn and BLASTx search of the two contig databases with a cut-off E-value of 1.0E^− 5^ against the NCBI nt/nr database identified 15,308 one to one (1:1) orthologous contigs, 70,493 *H. armigera*- and 30,239 *H. assulta*-specific contigs (Fig. 1; see the corresponding sheets in Dataset S1). The number of *H. armigera*-specific contigs was 2.33 times more than that of *H. assulta*-specific contigs (χ² test, *P* < 0.05), demonstrating that the transcriptome size of the larval labial salivary gland was significantly larger in *H. armigera* than in *H. assulta*.

**Table 1:** Summary statistics of the RNA-seq data of the labial salivary glands of *H. armigera* and *H. assulta*

Total number of reads	*H. armigera*	*H. assulta*
	38,149,826	36,178,424
Total assembled bases	51,412,837	46,013,942
GC content	41.37%	40.95%
Total number of contigs	85,801	45,547
N50 of contigs	877	1852
N90 of contigs	256	378
Mean contig size	596	1008
Total number of unigenes	69,340	36,345
Mapped rates of reads to contigs	76.82%	92.47%

### *H. armigera */ *H. assulta *size ratios of the GO terms and KEGG pathways related to the main functions of the larval labial salivary glands

#### *H. armigera* / *H. assulta* size ratios of the main GO functional terms

**Fig. 2:**
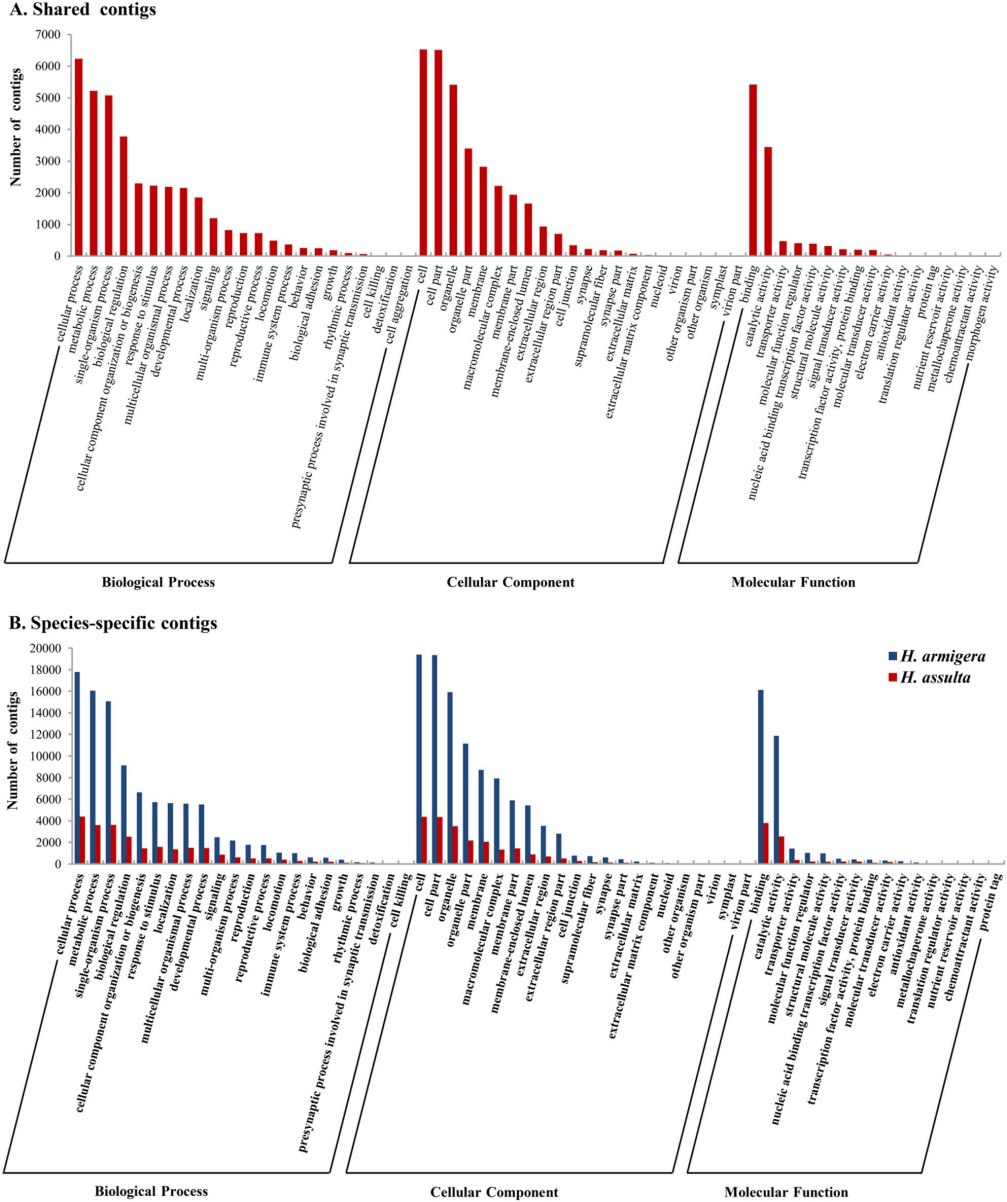
Gene ontology (GO) Level 2 functionary classification of the shared (**A**) and species-specific (**B**) contigs in the labial salivary gland of *H. armigera* and *H. assulta*

Gene ontology (GO) Level 2 functionary classification of the shared 1:1 orthologues and species-specific contigs in the labial salivary glands of *H. armigera* and *H. assulta* showed that the rankings of GO Level 2 functional categories based on the number of contigs in each category (i.e. the size of each category) were largely the same between the shared (Fig. 2A) and species-specific (Fig. 2B) contigs. In each GO category, there were always more *H. armigera*-specific contigs than *H. assulta*-specific contigs (Fig. 2B). The higher the ranking of the GO category, the greater the difference in the number of contigs within that category between the two species (Fig. 2B). Consistent with this trend, the sizes of 35 selected GO terms related to the major functions of labial salivary glands, such as synthesis and secretion of proteins, preoral/oral digestion of nutrients and detoxification metabolism, were significantly larger in the *H. armigera*-specific contigs than in the *H. assulta*-specific contigs, with a *H. armigera / H. assulta* ratio of 4.4 for the detoxification–related GO:0016787 (hydrolase activity) to 52.5 for the protein translation-related GO:0017101 (aminoacyl-tRNA synthetase multienzyme complex) (Table 2). All 35 selected GO terms were significantly enriched in the *H. armigera*-specific contigs, while only two of them, including GO:0003824 (catalytic activity) and GO:0016787 (hydrolase activity), were significantly enriched in the *H. assulta*-specific contigs (Table 2). These results disclosed that the *H. armigera / H. assulta* ratio of the labial transcriptome size was not equal across different functions, but was biased towards the tissue-specific functions crucial for the larval labial salivary glands.

**Table 2:** Interspecies size ratios and enrichment levels of 35 selected GO terms related to the major functions of the labial salivary gland of *H. armigera* and *H. assulta*

GO ID		*H. armigera*-specific		*H. assulta*-specific		*H. armigera */ *H. assulta* contig ratio	Shared	
	Description	Number of contigs	*P*-value	Number of contigs	*P*-value		Number of contigs	*P*-value
Cellular Component								
GO:0000502	proteasome complex	607	0.000000	26	0.985055	23.4	61	0.025992
GO:0031988	membrane-bounded vesicle	3166	0.000000	560	0.211166	5.7	787	0.805384
GO:0005798	Golgi-associated vesicle	310	0.000000	33	0.241626	9.4	40	0.825769
GO:0005783	endoplasmic reticulum	2470	0.000000	414	0.367870	5.97	594	0.657017
GO:1,990,904	ribonucleoprotein complex	2130	0.000000	263	0.999998	8.1	537	0.000002
GO:0030684	Preribosome	184	0.000318	15	0.954044	12.3	35	0.083006
GO:0017101	aminoacyl-tRNA synthetase multienzyme complex	105	0.000000	2	0.933648	52.5	7	0.221971
GO:0009328	phenylalanine-tRNA ligase complex	47	0.000785	2	0.672075	23.5	3	0.673496
GO:0044431	Golgi apparatus part	1172	0.003487	246	0.146896	4.8	330	0.872086
Biological Process								
GO:0019752	carboxylic acid metabolic process	2090	0.000000	273	0.514443	7.7	386	0.517936
GO:0072350	Tricarboxylic acid metabolic process	336	0.000000	24	0.552716	14	34	0.553407
GO:0005975	carbohydrate metabolic process	1669	0.000000	250	0.644042	6.7	363	0.387768
GO:1,901,564	organonitrogen compound metabolic process	3555	0.000000	466	0.999973	7.6	820	0.000035
GO:0019538	protein metabolic process	6404	0.000003	1302	0.962219	4.9	1944	0.041307
GO:0044281	small molecule metabolic process	3778	0.000000	584	0.783195	6.5	858	0.233868
GO:0022613	ribonucleoprotein complex biogenesis	1261	0.000000	92	0.999997	13.7	222	0.000005
GO:0006418	tRNA aminoacylation for protein translation	519	0.000000	16	0.999547	32.4	53	0.001141
GO:0006412	Translation	1436	0.000000	106	1.000000	13.6	311	0.000000
GO:0055114	oxidation-reduction process	2351	0.000000	337	0.950306	6.98	531	0.057512
GO:0098754	Detoxification	60	0.001656	2	0.859256	30	5	0.389289
Molecular Function								
GO:0000166	nucleotide binding	5766	0.000000	1032	0.559849	5.6	1457	0.458151
GO:0036094	small molecule binding	5927	0.000000	1067	0.657678	5.6	1520	0.358949
GO:0003824	catalytic activity	11,863	0.000000	2554	0.013080	4.65	3441	0.988098
GO:0004812	aminoacyl-tRNA ligase activity	519	0.000000	16	0.999587	32.4	53	0.001047
GO:0050662	coenzyme binding	1027	0.000000	94	0.838851	10.9	149	0.195625
GO:0097367	carbohydrate derivative binding	4630	0.000000	851	0.453685	5.4	1189	0.565719
GO:0016817	hydrolase activity, acting on acid anhydrides	2120	0.000000	361	0.293319	5.9	488	0.730959
GO:0016491	oxidoreductase activity	1985	0.000000	278	0.907485	7.1	430	0.106296
GO:0008238	exopeptidase activity	459	0.000000	31	0.920865	14.8	58	0.116874
GO:0008135	translation factor activity, RNA binding	480	0.000000	24	0.999779	20	72	0.000500
GO:0016787	hydrolase activity	5243	0.000000	1183	0.000635	4.4	1486	0.999456
GO:0003743	translation initiation factor activity	352	0.000000	12	0.999973	29.3	52	0.000089
GO:0008812	choline dehydrogenase activity	88	0.000000	6	0.117887	14.7	3	0.968596
GO:0004180	carboxypeptidase activity	171	0.000050	10	0.949397	17.1	24	0.101141
GO:0004177	aminopeptidase activity	231	0.000000	16	0.728719	14.4	26	0.384745

#### *H. armigera* / *H. assulta* size ratios of the main functionary KEGG pathways

Since the key tissue-specific function of the larval labial salivary glands of these two species is to synthesize and secrete proteins/enzymes into saliva for pre-oral (in-planta) and oral modification, digestion, or detoxification of host plant structures, nutrients, and defense compounds, we compared the *H. armigera* / *H. assulta* size ratios of 15 selected KEGG pathways related to these functions in the shared and species-specific contigs (Table 3). The 6 proteins/enzyme synthesis and secretion-related pathways exhibited the highest *H. armigera* / *H. assulta* size ratios [from 7.58 for biosynthesis of amino acids (ko01230) to 70.75 for protein export (ko03060)], followed by the 6 nutrient digestion-related pathways [from 0.79 for vitamin digestion and absorption (ko04977) to 12.74 for fatty acid degradation (ko00071)], and the 3 allelochemical detoxification-related pathways from 4.60 for drug metabolism - cytochrome P450 (ko00982) to 5.04 for nicotinate and nicotinamide metabolism (ko00760)] (Table 4). (Table 3). Notably, five out of the 6 protein/enzymes synthesis and secretion-related pathways were significantly enriched (*P* < 0.05) in *H. armigera*-specific contigs, while only one of them were enriched (*P* < 0.05) in the *H. assulta*-specific contigs. Of the 6 pathways related to nutrient digestion, carbon metabolism (ko01200), fatty acid degradation (ko00071) and glycolysis / gluconeogenesis (ko00010) were enriched (*P* < 0.05) in *H. armigera*-specific contigs, whereas starch and sucrose metabolism (ko00500), alanine, aspartate and glutamate metabolism (ko00250), and vitamin digestion and absorption (ko04977) were enriched (*P* < 0.05) in *H. assulta*-specific and contigs (Table 3).

**Table 3:** Interspecies size ratio and enrichment levels of 15 selected KEGG pathways related to the major functions of the labial salivary gland of *H. armigera* and *H. assulta*

Pathway ID			*H. armigera*-specific		*H. assulta*-specific		*H. armigera* / *H. assulta* contig ratio	Shared	
	Pathway		Number of contigs	*P*-value	Number of contigs	*P*-value		Number of contigs	*P*-value
Protein synthesis and secretion									
ko04141	Protein processing in endoplasmic reticulum		1294	5.25E-20	139	0.003584	9.31	135	0.997545
ko03060	Protein export		283	3.79E-08	4	0.999584	70.75	23	0.002002
ko01230	Biosynthesis of amino acids		402	0.000114	53	0.104056	7.58	55	0.926937
ko03020	RNA polymerase		140	0.306798	6	0.999282	23.33	27	0.002682
ko03013	RNA transport		826	0.014076	71	0.999658	11.63	151	0.000571
ko00970	Aminoacyl-tRNA biosynthesis		584	6.16E-12	22	0.997212	26.55	56	0.005722
Digestion									
ko01200		Carbon metabolism	737	4.16E-12	62	0.407142	11.89	79	0.657902
ko00500		Starch and sucrose metabolism	150	0.486812	44	0.007047	3.41	33	0.996372
ko00250		Alanine, aspartate and glutamate metabolism	102	0.299621	27	0.009368	3.78	17	0.996042
ko04977		Vitamin digestion and absorption	15	0.999884	19	0.02805	0.79	12	0.988480
ko00071		Fatty acid degradation	293	0.000149	23	0.601818	12.74	32	0.506348
ko00010		Glycolysis / Gluconeogenesis	323	0.000398	34	0.477530	9.50	44	0.612626
Detoxification									
ko00982		Drug metabolism - cytochrome P450	92	0.691909	20	0.358295	4.60	23	0.748573
ko00983		Drug metabolism - other enzymes	201	0.223004	43	0.046199	4.67	39	0.971634
ko00760		Nicotinate and nicotinamide metabolism	116	0.186837	23	0.127386	5.04	21	0.925250

**Table 4:** The top 10 enriched Go terms for cellular component, biological process and molecular function of the shared and species-specific contigs in the labial salivary gland transcriptomes of *H. armigera* and *H. assulta*

*H. armigera*-specific				*H. assulta*-specific				Shared			
GO ID	Description	Number of contigs	*P*-value	GO ID	Description	Number of contigs	*P*-value	GO ID	Description	Number of contigs	*P*-value
Cellular Component											
GO:0000502	proteasome complex	607	0.000000	GO:0031224	intrinsic component of membrane	1175	0.000038	GO:0043233	organelle lumen	1626	0.000000
GO:0098796	membrane protein complex	1568	0.000000	GO:0016021	integral component of membrane	1149	0.000057	GO:0070013	intracellular organelle lumen	1626	0.000000
GO:0032991	macromolecular complex	7910	0.000000	GO:0019012	virion	21	0.000091	GO:0031974	membrane-enclosed lumen	1659	0.000000
GO:0005737	Cytoplasm	14,620	0.000000	GO:0044447	axoneme part	22	0.000118	GO:0044424	intracellular part	6057	0.000000
GO:0044444	cytoplasmic part	10,707	0.000000	GO:0030286	dynein complex	36	0.000160	GO:0031981	nuclear lumen	1387	0.000000
GO:0043234	protein complex	6327	0.000000	GO:0034703	cation channel complex	24	0.000177	GO:0005622	Intracellular	6160	0.000000
GO:0031988	membrane-bounded vesicle	3166	0.000000	GO:0044423	virion part	13	0.000323	GO:0043231	intracellular embrane-bounded organelle	4791	0.000000
GO:0044432	endoplasmic reticulum part	1853	0.000000	GO:1,990,752	microtubule end	13	0.000323	GO:0044428	nuclear part	1637	0.000000
GO:0031982	Vesicle	3271	0.000000	GO:0071944	cell periphery	913	0.000368	GO:0043227	membrane-bounded organelle	4960	0.000000
GO:0005783	endoplasmic reticulum	2470	0.000000	GO:0005858	axonemal dynein complex	16	0.000375	GO:0005840	Ribosome	205	0.000000
Biological Process											
GO:0019752	carboxylic acid metabolic process	2090	0.000000	GO:0006278	RNA-dependent DNA replication	348	0.000000	GO:0010467	gene expression	1964	0.000000
GO:0043436	oxoacid metabolic process	2172	0.000000	GO:0015074	DNA integration	229	0.000000	GO:0006412	Translation	311	0.000000
GO:0006082	organic acid metabolic process	2185	0.000000	GO:0006259	DNA metabolic process	686	0.000000	GO:0006518	peptide metabolic process	345	0.000000
GO:1,901,564	organonitrogen compound metabolic process	3555	0.000000	GO:0006260	DNA replication	406	0.000000	GO:0016070	RNA metabolic process	1737	0.000000
GO:0044281	small molecule metabolic process	3778	0.000000	GO:0032196	transposition	184	0.000000	GO:0043043	peptide biosynthetic process	319	0.000000
GO:0022613	ribonucleoprotein complex biogenesis	1261	0.000000	GO:0006313	transposition, DNA-mediated	155	0.000000	GO:0043603	cellular amide metabolic process	394	0.000000
GO:0006418	tRNA aminoacylation for protein translation	519	0.000000	GO:0090305	nucleic acid phosphodiester bond hydrolysis	298	0.000000	GO:0044271	cellular nitrogen compound biosynthetic process	1678	0.000000
GO:0043038	amino acid activation	519	0.000000	GO:0006310	DNA recombination	277	0.000000	GO:0043604	amide biosynthetic process	340	0.000000
GO:0043039	tRNA aminoacylation	519	0.000000	GO:0051881	regulation of mitochondrial membrane potential	36	0.000005	GO:0006413	translational initiation	75	0.000000
GO:1,901,566	organonitrogen compound biosynthetic process	2462	0.000000	GO:0051901	positive regulation of mitochondrial depolarization	16	0.000042	GO:0044267	cellular protein metabolic process	1627	0.000000
Molecular Function											
GO:0000166	nucleotide binding	5766	0.000000	GO:0003964	RNA-directed DNA polymerase activity	347	0.000000	GO:0003735	structural constituent of ribosome	166	0.000001
GO:1,901,265	nucleoside phosphate binding	5766	0.000000	GO:0034061	DNA polymerase activity	353	0.000000	GO:0003723	RNA binding	891	0.000028
GO:0036094	small molecule binding	5927	0.000000	GO:0016779	nucleotidyltransferase activity	375	0.000000	GO:0003743	translation initiation factor activity	52	0.000089
GO:0003824	catalytic activity	11,863	0.000000	GO:0004190	aspartic-type endopeptidase activity	169	0.000000	GO:0003899	DNA-directed RNA polymerase activity	39	0.000296
GO:0043168	anion binding	5559	0.000000	GO:0070001	aspartic-type peptidase activity	169	0.000000	GO:0016651	oxidoreductase activity, acting on NAD(P)H	40	0.000487
GO:0048037	cofactor binding	1208	0.000000	GO:0016772	transferase activity, transferring phosphorus-containing groups	699	0.000000	GO:0008135	translation factor activity, RNA binding	72	0.0005
GO:0032553	ribonucleotide binding	4454	0.000000	GO:0004519	endonuclease activity	237	0.000000	GO:0003677	DNA binding	1050	0.000813
GO:0004812	aminoacyl-tRNA ligase activity	519	0.000000	GO:0004518	nuclease activity	284	0.000000	GO:0000217	DNA secondary structure binding	13	0.00092
GO:0016875	ligase activity, forming carbon-oxygen bonds	519	0.000000	GO:0004175	endopeptidase activity	281	0.000000	GO:0004812	aminoacyl-tRNA ligase activity	53	0.001047
GO:0016876	ligase activity, forming aminoacyl-tRNA and related compounds	519	0.000000	GO:0070011	peptidase activity, acting on L-amino acid peptides	353	0.000001	GO:0016875	ligase activity, forming carbon-oxygen bonds	53	0.001047

### *H. armigera* / *H. assulta* size ratios of the top enriched GO terms and pathways of the larval labial salivary glands

#### Size ratio of the top 30 enriched Go terms

Further interspecies comparisons were conducted to clarify whether the most enriched GO terms in the labial salivary gland transcriptomes of *H. armigera* and *H. assulta* were completely different or partially overlapping (Table 4), and whether their size ratios of *H. armigera* / *H. assulta* were consistently greater than 1 (Table 5). No overlaps were found among the top 10 enriched GO terms in the categories of biological process, cellular component, and molecular function (a total of 30 GO terms) between *H. armigera*- and *H. assulta*-specific contigs, as well as between the shared contigs and *H. assulta*-specific contigs (Table 4). However, GO:0004812 **(**aminoacyl-tRNA ligase activity) and GO:0016875 **(**ligase activity, forming carbon-oxygen bonds), two terms basically referring to the same group of ligases that join various tRNA molecules and amino acids, an essential step for protein translation, were enlisted among the top 30 GO terms of both *H. armigera*-specific and shared contigs (Table 4). The top 30 GO terms of *H- armigera*-specific contigs were largely involved in protein translation (GO:0026163, GO:0006418, GO:0043038, GO:0043039, GO:1901566, GO:0004812, GO:0016875, GO:0016876), protein modification/transport/secretion (GO:0098796, GO:0031988, GO:0044432, GO:0031982, GO:0005783), metabolism of acids / organonitrogen / small molecules (GO:0019752, GO:0043436, GO:0006082, GO:1901564, GO:0044281), and binding of nucleotide / ribonucleotide / nucleoside phosphate / small molecule / anion / cofactor (GO:0000166, GO:1901265, GO:0036094, GO:0043168, GO:0048037, GO:0032553). By contrast, the top 30 GO terms of *H. assulta*-specific contigs were largely associated with DNA replication/repair (GO:0006259, GO:0006260, GO:0090305, GO:0006310, GO:0034061, GO:0016779, GO:0004519, GO:0004518), microtubule-based cytoskeletal structures for cargo transport /motility (GO:0044447, GO:0030286, GO:1990752, GO:0071944, GO:0005858), digestion of protein nutrients (GO:0004190, GO:0070001, GO:0004175, GO:0070011), membrane proteins/receptors (GO:0031224, GO:0016021, GO:0016772, GO:0034703), and mitochondrial potential regulation (GO:0051881, GO:0051901). Notably, five of the top 30 GO terms of *H. assulta*-specific contigs were related to transposition of transposable elements (TEs) (GO:0006278, GO:0015074, GO:0032196, GO:0006313, GO:0003964), rather than to the normal functions of insect cells (Table 4).

**Table 5:** Interspecies size ratios of the top 30 enriched GO terms for *H. armigera* and *H. assulta* and their enrichment statuses in the other specie

GO ID		*H. armigera* top GO terms		Status in *H. assulta*		*H. armigera* / *H. assulta* contig ratio
	Description	Number of contigs	*P*-value	Number of contigs	*P*-value	
Cellular Component						
GO:0000502	proteasome complex	607	0.000000	26	0.985055	23.35
GO:0098796	membrane protein complex	1568	0.000000	230	0.008014	6.82
GO:0032991	macromolecular complex	7910	0.000000	1321	0.999998	5.99
GO:0005737	Cytoplasm	14,620	0.000000	2872	0.999854	5.09
GO:0044444	cytoplasmic part	10,707	0.000000	1970	0.999628	5.44
GO:0043234	protein complex	6327	0.000000	1064	0.999037	5.95
GO:0031988	membrane-bounded vesicle	3166	0.000000	560	0.211166	5.65
GO:0044432	endoplasmic reticulum part	1853	0.000000	312	0.100026	5.94
GO:0031982	Vesicle	3271	0.000000	577	0.339759	5.67
GO:0005783	endoplasmic reticulum	2470	0.000000	414	0.367870	5.97
Biological process						
GO:0019752	carboxylic acid metabolic process	2090	0.000000	273	0.514443	7.66
GO:0043436	oxoacid metabolic process	2172	0.000000	295	0.499554	7.36
GO:0006082	organic acid metabolic process	2185	0.000000	299	0.617340	7.31
GO:1,901,564	organonitrogen compound metabolic process	3555	0.000000	466	0.999973	7.63
GO:0044281	small molecule metabolic process	3778	0.000000	584	0.783195	6.47
GO:0022613	ribonucleoprotein complex biogenesis	1261	0.000000	92	0.999997	13.71
GO:0006418	tRNA aminoacylation for protein translation	519	0.000000	16	0.999547	32.44
GO:0043038	amino acid activation	519	0.000000	16	0.999547	32.44
GO:0043039	tRNA aminoacylation	519	0.000000	16	0.999547	32.44
GO:1,901,566	organonitrogen compound biosynthetic process	2462	0.000000	315	0.999667	7.82
Molecular function						
GO:0000166	nucleotide binding	5766	0.000000	1032	0.559849	5.59
GO:1,901,265	nucleoside phosphate binding	5766	0.000000	1032	0.559849	5.59
GO:0036094	small molecule binding	5927	0.000000	1067	0.657678	5.56
GO:0003824	catalytic activity	11,863	0.000000	2554	0.013080	4.65
GO:0043168	anion binding	5559	0.000000	1011	0.442827	5.50
GO:0048037	cofactor binding	1208	0.000000	118	0.883033	8.71
GO:0032553	ribonucleotide binding	4454	0.000000	813	0.335932	5.48
GO:0004812	aminoacyl-tRNA ligase activity	519	0.000000	16	0.999587	32.44
GO:0016875	ligase activity, forming carbon-oxygen bonds	519	0.000000	16	0.999587	32.44
GO:0016876	ligase activity, forming aminoacyl-tRNA and related compounds	519	0.000000	16	0.999587	32.44
		*H. assulta top GO terms*		Status in *H. armigera*		
Cellular component						
GO:0031224	intrinsic component of membrane	1175	0.000038	4094	0.998060	3.48
GO:0016021	integral component of membrane	1149	0.000057	4044	0.992858	3.52
GO:0019012	Virion	21	0.000091	21	0.979368	1.0
GO:0044447	axoneme part	22	0.000118	28	0.086094	1.27
GO:0030286	dynein complex	36	0.000160	111	0.009234	3.08
GO:0034703	cation channel complex	24	0.000177	28	0.367642	1.17
GO:0044423	virion part	13	0.000323	12	0.880953	0.92
GO:1,990,752	microtubule end	13	0.000323	29	0.138065	2.23
GO:0071944	cell periphery	913	0.000368	2879	1.000000	3.15
GO:0005858	axonemal dynein complex	16	0.000375	24	0.160027	1.5
Biological process						
GO:0006278	RNA-dependent DNA replication	348	0.000000	244	1.000000	0.70
GO:0015074	DNA integration	229	0.000000	185	0.999997	0.81
GO:0006259	DNA metabolic process	686	0.000000	1248	1.000000	1.82
GO:0006260	DNA replication	406	0.000000	612	1.000000	1.51
GO:0032196	Transposition	184	0.000000	111	1.000000	0.60
GO:0006313	transposition, DNA-mediated	155	0.000000	86	1.000000	0.56
GO:0090305	nucleic acid phosphodiester bond hydrolysis	298	0.000000	511	0.999998	1.72
GO:0006310	DNA recombination	277	0.000000	339	1.000000	1.22
GO:0051881	regulation of mitochondrial membrane potential	36	0.000005	53	0.250731	1.47
GO:0051901	positive regulation of mitochondrial depolarization	16	0.000042	11	0.176575	0.69
Molecular function						
GO:0003964	RNA-directed DNA polymerase activity	347	0.000000	243	1.000000	0.70
GO:0034061	DNA polymerase activity	353	0.000000	291	1.000000	0.82
GO:0016779	nucleotidyltransferase activity	375	0.000000	569	1.000000	1.52
GO:0004190	aspartic-type endopeptidase activity	169	0.000000	157	0.999689	0.93
GO:0070001	aspartic-type peptidase activity	169	0.000000	157	0.999689	0.93
GO:0016772	transferase activity, transferring phosphorus-containing groups	699	0.000000	1473	1.000000	2.11
GO:0004519	endonuclease activity	237	0.000000	334	0.999927	1.41
GO:0004518	nuclease activity	284	0.000000	467	0.999999	1.64
GO:0004175	endopeptidase activity	281	0.000000	809	0.020479	2.88
GO:0070011	peptidase activity, acting on L-amino acid peptides	353	0.000001	1350	0.000001	3.82

Table 5 shows that only two [GO:0098796 **(**membrane protein complex) and GO:0003824 **(**catalytic activity)] of the top 30 enriched GO terms of *H. armigera* were enriched (*P* < 0.05) in *H. assulta*-specific contigs. By contrast, three [GO:0030286 **(**dynein complex), GO:0004175 **(**endopeptidase activity) and GO:0070011 **(**peptidase activity, acting on L-amino acid peptides)] of the top 30 enriched GO terms of *H. assulta* were also overrepresented (*P* < 0.05) in *H. armigera*-specific contigs (Table 5). Moreover, the *H. armigera* / *H. assulta* ratios of the sizes for all the top 30 GO terms of *H. armigera* ranged from 4.65 (catalytic activity, GO:0003824) to 32.44 (aminoacyl-tRNA ligase activity, GO:0004812). Counterintuitively, 23 of the 30 top enriched GO terms of *H. assulta* also had a *H. armigera* / *H. assulta* size ratio of 1 or larger (0.92–3.82). Only two (GO:0051901, GO:0034061) of the remaining 7 *H. assulta* GO terms with a *H. armigera* / *H. assulta* size ratio < 0.92 (namely significantly less than 1) were involved in the normal function of insect cells, whereas the other 5 terms (GO:0006278, GO:0015074, GO:0032196, GO:0006313, GO:0003964) were associated with transposition of TEs (Table 5), indicating that significantly more self-movable TEs were transcribed in *H. assulta* than in *H. armigera*.

#### Size ratio of the top enriched KEGG pathways

Similar interspecies comparisons were conducted to reveal if the most enriched KEGG pathways in the labial salivary gland transcriptomes of *H. armigera* and *H. assulta* were also largely different (Table 6), and whether most of the most enriched pathways of either species, like their top enriched GO terms (Table 5), also had a size ratio of *H. armigera* / *H. assulta* equal to or greater than 1 (Table 7). The shared, *H. armigera*- and *H. assulta*-specific contigs were significantly enriched in 21, 32 and 28 KEGG pathways (see the pathway enrichment sheets in Dataset S1), respectively. While no overlap was found in the top 20 enriched pathways between the *H. assulta*-specific and shared contigs, four pathways including oxidative phosphorylation (ko00190), protein export (ko03060), aminoacyl-tRNA biosynthesis (ko00970) and proteasome (ko03050) were among the top 20 enriched pathways of both the *H. armigera*-specific and shared contigs (Table 6). Only the pathway ko04141 (protein processing in endoplasmic reticulum) was among the top 20 enriched pathways of both *H. armigera*- and *H. assulta*-specific contigs. None of the other 19 top enriched pathways of the *H. armigera*-specific contigs were overrepresented in the *H. assulta*-specific contigs, and vice versa (Table 6). *H. armigera*-specific contigs were mainly enriched in the pathways related to protein synthesis /secretion (ko03040, ko00970, ko03008, N ko00510, ko03060, ko03050, ko04141) and metabolism of nutrients (e.g. ko01230, ko00260, ko00380, ko00410, ko01200, ko00010, ko00020, ko00071, ko00010, ko00062, ko00860). By contrast, *H. assulta*-specific contigs were just concentrated in the pathways related to biological clock (ko04710, ko04711, 53) and signaling transduction (ko04080, ko03320, ko04620, ko04015, ko04668) (Table 6). The size ratios of *H. armigera* / *H. assulta* for the top 20 enriched pathways of *H. armigera* extended from 6.94 for ko00062 (fatty acid elongation) to 70.75 for ko03060 (protein export) (Table 7). Other than ko04977 (vitamin digestion and absorption) and ko04711 (circadian rhythm-fly), which had a size ratio of 0.79 and 0.93, respectively, the size ratios of the other 18 top enriched pathways of *H. assulta* were greater than 1, varying between 1.23 for ko04713 (circadian entrainment) and 9.31 for ko04141 (protein processing in endoplasmic reticulum) (Table 7).

**Table 6:** The top 20 enriched KEGG pathways of species-specific and shared contigs in the labial salivary gland transcriptomes of *H. armigera* and *H. assulta*

*H. armigera*-specific				*H. assulta*-specific				Shared			
Pathway ID	**Pathway**	**Number of contigs**	**P-value**	**Pathway ID**	**Pathway**	**Number of contigs**	**P-value**	**Pathway ID**	**Pathway**	**Number of contigs**	**P-value**
ko04141	Protein processing in endoplasmic reticulum	1294	5.25E-20	ko04710	Circadian rhythm	34	3.43E-05	ko03010	Ribosome	139	8.24E-06
ko03050	Proteasome	533	1.13E-15	ko04711	Circadian rhythm - fly	30	0.000147	ko05012	Parkinson’s disease	98	1.14E-05
ko01200	Carbon metabolism	737	4.16E-12	ko04080	Neuroactive ligand-receptor interaction	24	0.000203	ko00190	Oxidative phosphorylation	98	0.00015
ko00970	Aminoacyl-tRNA biosynthesis	584	6.16E-12	ko04713	Circadian entrainment	53	0.00028	ko03013	RNA transport	151	0.000571
ko00510	N-Glycan biosynthesis	454	1.29E-11	ko03320	PPAR signaling pathway	55	0.000295	ko03060	Protein export	23	0.002002
ko00020	Citrate cycle (TCA cycle)	353	7.44E-09	ko04510	Focal adhesion	114	0.000596	ko03030	DNA replication	42	0.00251
ko03060	Protein export	283	3.79E-08	ko04620	Toll-like receptor signaling pathway	40	0.000654	ko03020	RNA polymerase	27	0.002682
ko03008	Ribosome biogenesis in eukaryotes	539	7.98E-06	ko04145	Phagosome	81	0.002375	ko05169	Epstein-Barr virus infection	122	0.003106
ko00260	Glycine, serine and threonine metabolism	315	1.86E-05	ko04540	Gap junction	56	0.002542	ko05010	Alzheimer’s disease	105	0.003375
ko01230	Biosynthesis of amino acids	402	0.000114	ko04141	Protein processing in endoplasmic reticulum	139	0.003584	ko03430	Mismatch repair	23	0.005125
ko00071	Fatty acid degradation	293	0.000149	ko04015	Rap1 signaling pathway	98	0.004971	ko00970	Aminoacyl-tRNA biosynthesis	56	0.005722
ko00010	Glycolysis / Gluconeogenesis	323	0.000398	ko04668	TNF signaling pathway	41	0.005091	ko04350	TGF-beta signaling pathway	43	0.005844
ko01210	2-Oxocarboxylic acid metabolism	169	0.000499	ko04380	Osteoclast differentiation	32	0.006854	ko00061	Fatty acid biosynthesis	20	0.0067
ko03040	Spliceosome	767	0.000632	ko00500	Starch and sucrose metabolism	44	0.007047	ko04932	Non-alcoholic fatty liver disease (NAFLD)	86	0.007275
ko00860	Porphyrin and chlorophyll metabolism	243	0.001505	ko04611	Platelet activation	71	0.007631	ko03050	Proteasome	38	0.008181
ko04721	Synaptic vesicle cycle	233	0.002138	ko00250	Alanine, aspartate and glutamate metabolism	27	0.009368	ko04340	Hedgehog signaling pathway	28	0.008552
ko00062	Fatty acid elongation	118	0.003836	ko04962	Vasopressin-regulated water reabsorption	39	0.010006	ko04110	Cell cycle	102	0.010613
ko00190	Oxidative phosphorylation	609	0.004634	ko05146	Amoebiasis	52	0.018616	ko04614	Renin-angiotensin system	13	0.016614
ko00380	Tryptophan metabolism	165	0.004773	ko05132	Salmonella infection	53	0.022968	ko05217	Basal cell carcinoma	33	0.031456
ko00410	beta-Alanine metabolism	188	0.005012	ko04977	Vitamin digestion and absorption	19	0.02805	ko04260	Cardiac muscle contraction	33	0.031456

**Table 7:** Interspecies size ratios of the top 20 enriched KEGG pathways for *H. armigera* and *H. assulta* and their enrichment statuses in the other species

Pathway ID		*H. armigera* top KEGG pathways		Status in *H. assulta*		*H. armigera* / *H. assulta* contig ratio
	Description	Number of contigs	*P*-value	Number of contigs	*P*-value	
ko04141	Protein processing in endoplasmic reticulum	1294	5.25E-20	139	0.003584	9.309352518
ko03050	Proteasome	533	1.13E-15	13	0.996591	41
ko01200	Carbon metabolism	737	4.16E-12	62	0.407142	11.88709677
ko00970	Aminoacyl-tRNA biosynthesis	584	6.16E-12	22	0.997213	26.54545455
ko00510	N-Glycan biosynthesis	454	1.29E-11	33	0.194521	13.75757576
ko00020	Citrate cycle (TCA cycle)	353	7.44E-09	23	0.601818	15.34782609
ko03060	Protein export	283	3.79E-08	4	0.999584	70.75
ko03008	Ribosome biogenesis in eukaryotes	539	7.98E-06	54	0.517063	9.981481481
ko00260	Glycine, serine and threonine metabolism	315	1.86E-05	26	0.463026	12.11538462
ko01230	Biosynthesis of amino acids	402	0.000114	53	0.104056	7.58490566
ko00071	Fatty acid degradation	293	0.000149	23	0.601818	12.73913043
ko00010	Glycolysis / Gluconeogenesis	323	0.000398	34	0.47753	9.5
ko01210	2-Oxocarboxylic acid metabolism	169	0.000499	19	0.18246	8.894736842
ko03040	Spliceosome	767	0.000632	90	0.727145	8.522222222
ko00860	Porphyrin and chlorophyll metabolism	243	0.001505	29	0.297195	8.379310345
ko04721	Synaptic vesicle cycle	233	0.002138	18	0.887897	12.94444444
ko00062	Fatty acid elongation	118	0.003836	17	0.060961	6.941176471
ko00190	Oxidative phosphorylation	609	0.004634	37	0.999928	16.45945946
ko00380	Tryptophan metabolism	165	0.004773	21	0.135583	7.857142857
ko00410	beta-Alanine metabolism	188	0.005012	20	0.216771	9.4
		*H. assulta top* KEGG pathways		Status in *H. armigera*		
ko04710	Circadian rhythm	34	3.43E-05	60	0.455921	1.764705882
ko04711	Circadian rhythm - fly	30	0.000147	28	0.933051	0.933333333
ko04080	Neuroactive ligand-receptor interaction	24	0.000203	46	0.237797	1.916666667
ko04713	Circadian entrainment	53	0.00028	65	0.999221	1.226415094
ko03320	PPAR signaling pathway	55	0.000295	149	0.822809	2.709090909
ko04510	Focal adhesion	114	0.000596	282	0.999948	2.473684211
ko04620	Toll-like receptor signaling pathway	40	0.000654	61	0.969385	1.525
ko04145	Phagosome	81	0.002375	382	0.05738	4.716049383
ko04540	Gap junction	56	0.002542	128	0.99277	2.285714286
ko04141	Protein processing in endoplasmic reticulum	139	0.003584	1294	5.25E-20	9.309352518
ko04015	Rap1 signaling pathway	98	0.004971	168	1	1.714285714
ko04668	TNF signaling pathway	41	0.005091	117	0.808904	2.853658537
ko04380	Osteoclast differentiation	32	0.006854	89	0.845974	2.78125
ko00500	Starch and sucrose metabolism	44	0.007047	150	0.486812	3.409090909
ko04611	Platelet activation	71	0.007631	202	0.931609	2.845070423
ko00250	Alanine, aspartate and glutamate metabolism	27	0.009368	102	0.299621	3.777777778
ko04962	Vasopressin-regulated water reabsorption	39	0.010006	153	0.096715	3.923076923
ko05146	Amoebiasis	52	0.018616	96	0.999786	1.846153846
ko05132	Salmonella infection	53	0.022968	193	0.806551	3.641509434
ko04977	Vitamin digestion and absorption	19	0.02805	15	0.999884	0.789473684

### *H. armigera* / *H. assulta* differences in contigs inserted with TEs in their labial salivary gland transcriptomes as well as in TE content and pseudogenized detoxification gene numbers in their genomes

**Fig. 3:**
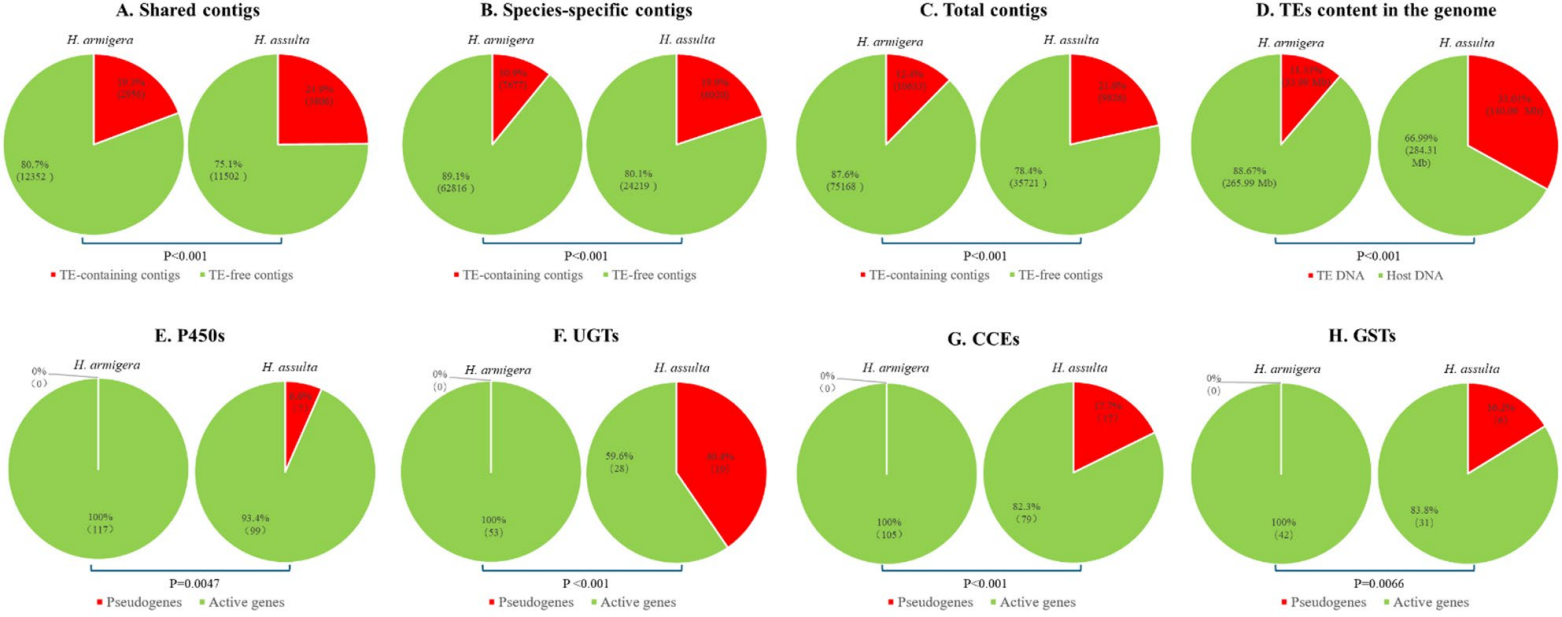
Interspecies comparison of TE-containing contigs in the shared (**A**), species-specific (**B**) and total contigs (**C**) of the liable salivary gland transcriptomes of *H. armigera* and *H. assulta*, as well as of the TE content (**D**) and the pseudogenized members of the P450 (**E**), UGT (**F**), CCE (**G**) and GST (**H**) families in the genomes of these two species. For each of A-C composite figures, red color = TE-containing contigs, green color = TE-free contigs. For composite figure D, red color = TE DNA, green color = host DNA. For each of E-H composite figures, red color = pseudogenes, Green color = active genes. Chi-square tests are performed to test if the left and right pies of each composite figure are significantly different from each other. The percentages of TE contents in the genomes of the two species were from Kim et al. (2024) [67] and Zhang et al. (2023) [66], respectively. The numbers of active and pseudogenized members of the four detoxification gene families used to make the composite figures E-H were from Kim et al. (2024) [67] and Zhang et al. (2024) [68]

To confirm whether TEs in the specialist *H. assulta* and the generalist *H. armigera* undergo different degrees of activation and transposition frequencies in accordance with their differences in the enrichment status and size of GO terms associated TE transposition in their labial salivary glands transcriptomes (Tables 4 and 5), we performed a chi-square test comparing the percentage of TE-containing contigs in their labial slaviary gland transcriptomes, as well as the TE content and the percentage of pseudogenized detoxification genes in their genomes (Fig. 3). Compatible with their difference in the enrichment status and size of transposition-related GO terms (Tables 4 and 5), significantly more TE-containing contigs were found in the shared (Fig. 3A), species-specific (Fig. 3B), and total contigs (Fig. 3C) of *H. assulta* labial salivary gland transcriptome, compared to the corresponding contigs of *H. armigera* labial salivary gland transcriptome. Along the same line, the percentages of TE contents (Fig. 3D) and of pseudogenes in the families of P450 (Fig. 3E), UGT (Fig. 3F), CCE (Fig. 3G) and GST (Fig. 3H) were all significantly higher in *H. assulta* than in *H. armigera.*

### *H. armigera / H. assulta differences* in the numbers of genes and alleles expressed in their labial salivary glands

**Fig. 4:**
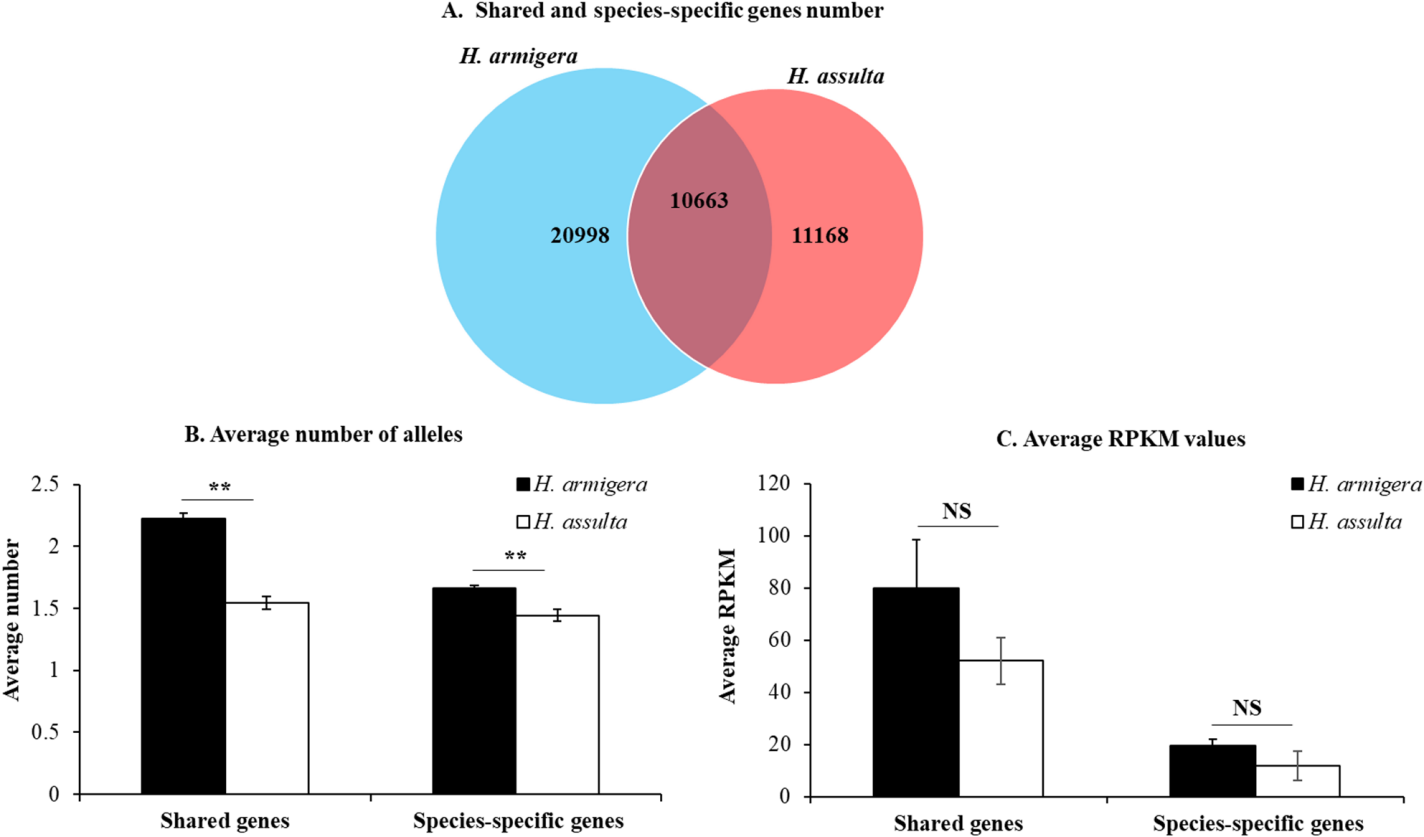
Venn diagram of the shared and species-specific genes (**A**) expressed in labial salivary gland of *H. armigera* and *H. assulta* and their average number of alleles (**B**) and average RPKM values (**C**) in the two species. The lists of the shared and species-specific genes in (**A**) can be found in the corresponding sheets of Dataset S1. The data and error bars in (**B**-**C**) represent the means and standard errors. Significant (Independent t-test, *P* < 0.05) and extremely significant (Independent t-test, *P* < 0.01) differences between the two species are indicated by “*” and “**”, respectively. NS = no significant difference

To verify that the labial salivary transcriptome size of *H. armigera* is also larger than that of *H. assulta* not only at the contig level (Sect.  3.1–3.4) but also at the gene and allele levels (hereafter), we further clustered the shared and species-specific contigs (Fig. 1) into shared/species-specific genes and alleles using the corresponding criteria described in Sect.  2.5. This led to identification of 10,663 shared orthologous genes, 20,998 *H. armigera*-specific genes, and 11,168 *H. assulta*-specific genes (Fig. 4A). Similar to the contig level, the number of expressed genes specific to *H. armigera* was 1.88 times (20998/11168) that of genes specific to *H. assulta* (χ² test, *P* < 0.05), further demonstrating that the transcriptome size of the larval labial salivary gland was significantly larger in *H. armigera* than in *H. assulta*. Moreover, the average number of alleles for each shared and species-specific gene in *H. armigera* was significantly greater than that in *H. assulta* (Fig. 4B). However, the average expression level (RPKM value) of the shared genes and species-specific genes were only numerically higher (not significant) in *H. armigera* than in *H. assulta* (Fig. 4C).

### *H. armigera* / *H. assulta* size ratio of the environmental and essential genes / alleles expressed in their labial salivary glands

**Fig. 5:**
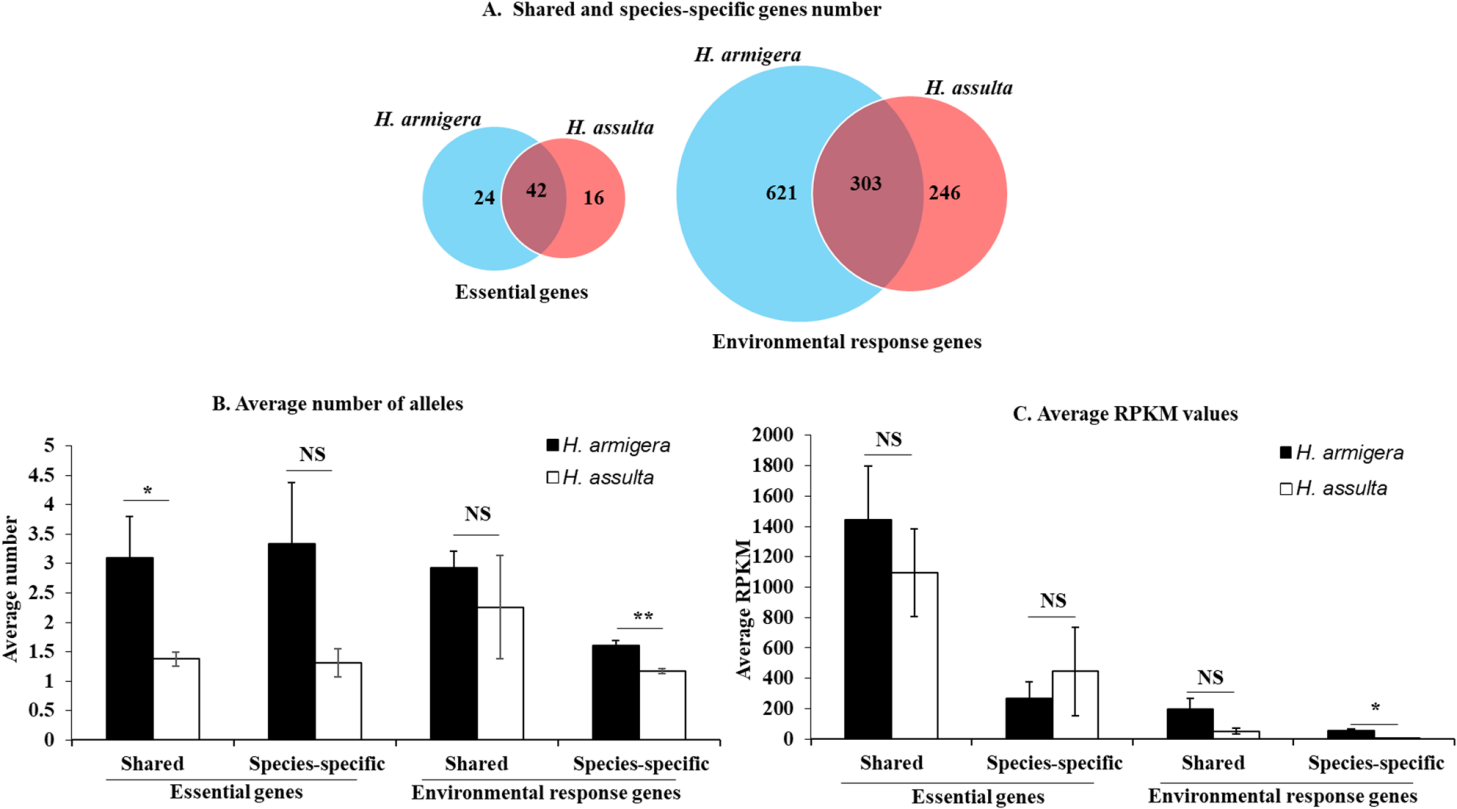
Venn diagram of the shared and species-specific essential and environment response genes (**A**) expressed in labial salivary gland of *H. armigera* and *H. assulta* and their average number of alleles (**B**) and average RPKM values (**C**) in the two species. The lists of the shared and species-specific essential and environment response genes in (**A**) can be found in the corresponding sheets of Dataset S1. The data and error bars in (**B-C**) represent the means and standard errors. Significant (Independent t-test, *P* < 0.05) and extremely significant (Independent t-test, *P* < 0.01) differences between the two species are indicated by “*” and “**”, respectively. NS = no significant difference

To test if the *H. armigera / H. assulta* ratio of the labial transcriptome size was biased towards the environment response genes (refers to genes that are involved in environmental adaptation), relative to the essential genes (refers to housekeeping genes that indispensable for the survival, growth and reproduction of an organism), we used a subset of 208 selected environment response genes and a total of 606 known *D. melanogaster* essential genes (see the essential and environment response gene sheets in Dataset S1) to identify the corresponding *H. armigera* and *H. assulta* essential and environment response genes. The number of the shared essential genes (42) by the two species was about the same with that of the species-specific essential genes (*H. armigera* 24 + *H. assulta* 16 = 40) (Fig. 5A). By contrast, the number of the shared environment response genes (303) was 2.86 times less than that of species-specific environment response genes (621 + 246 = 867) (Fig. 5A). The number of *H. armigera*-specific essential genes was only 1.5 times more than that of *H. assulta*-specific essential genes, whereas the number of *H. armigera*-specific environment response genes was 2.52 times that of *H. assulta*-specific environment response genes (Fig. 5A). In terms of the number of alleles per gene, *H. armigera* had numerically more alleles for species-specific essential genes and shared environmental response genes and significantly more alleles for shared essential genes and species-specific environmental response genes than did *H. assulta* (Fig. 5B). The average gene expression level was also numerically higher for shared essential and environmental genes and significantly higher for species-specific environmental genes in *H. armigera* than in *H. assulta* (Fig. 5C).

### *H. armigera* / *H. assulta* differences in the numbers of putative secreted protein genes and alleles expressed in their labial salivary glands

**Fig. 6:**
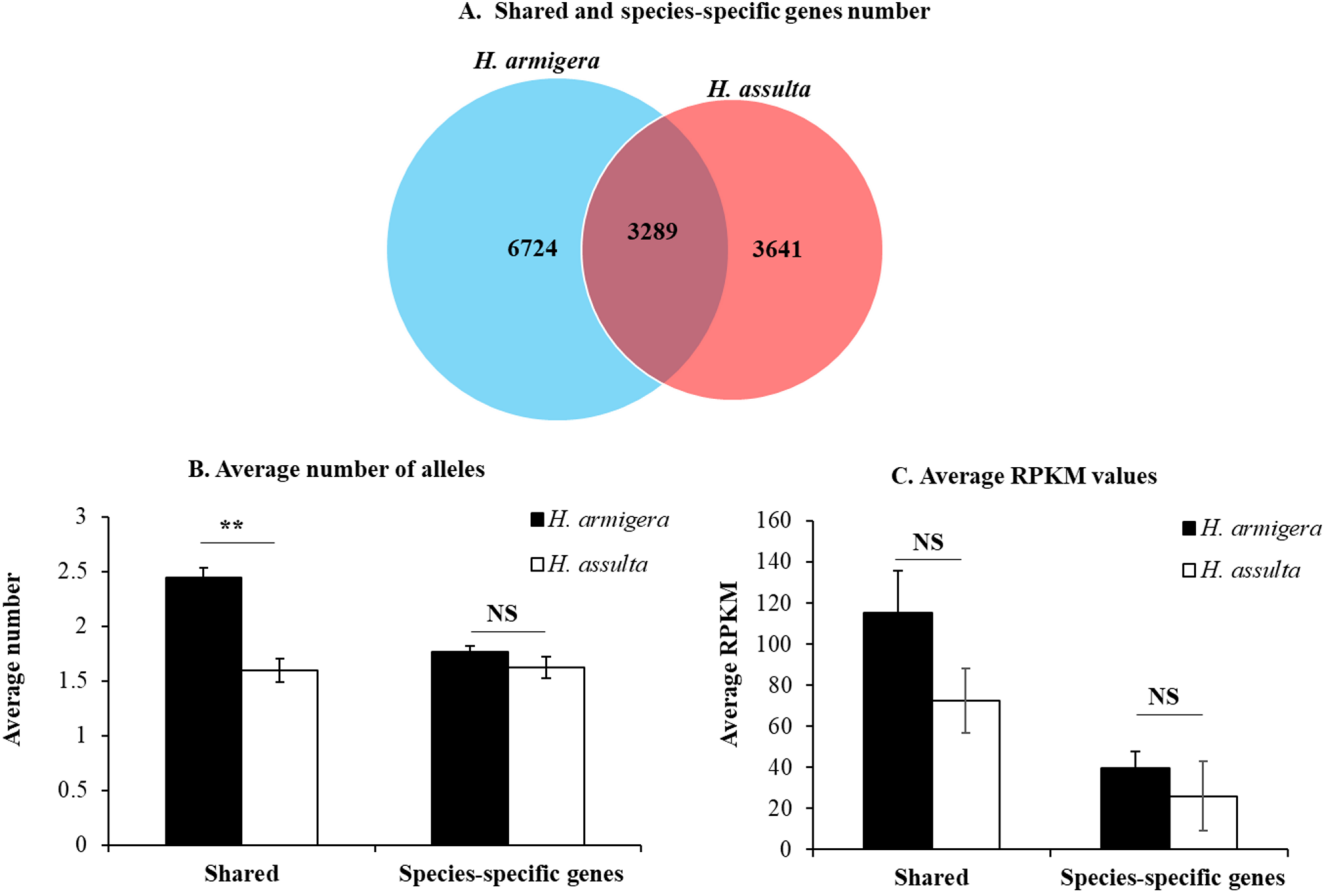
Venn diagram of the shared and species-specific putative secreted protein genes (**A**) expressed in labial salivary gland of *H. armigera* and *H. assulta* and their average number of alleles (**B**) and average RPKM values (**C**) in the two species. The lists of the shared and species-specific putative secreted protein genes in (**A**) can be found in the corresponding sheets of Dataset S1. The data and error bars in (**B-C**) represent the means and standard errors. Significant (Independent t-test, *P* < 0.05) and extremely significant (Independent t-test, *P* < 0.01) differences between the two species are indicated by “*” and “**”, respectively. NS = no significant difference

To test if the labial salivary gland secretome size (the number of putative secreted protein genes) of the generalist *H. armigera* is larger than that of its specialist sibling *H. assulta*, we bioinformatically identified all the putative secreted protein genes from the labial salivary gland transcriptomes of the two species. Other than sharing 3289 putative secreted protein genes with *H. assulta*, *H. armigera* also had 6724 species-specific putative secreted protein genes, which was 1.85 times that of *H. assulta* (Fig. 6A). The number of alleles per putative secreted protein gene was numerically higher for species-specific ones and significantly higher for shared ones in *H. armigera* than *H. assulta* (Fig. 6B). The average expression level (RPKM value) of putative secreted protein genes was also numerically higher for both shared and species-specific ones in *H. armigera* than in *H. assulta* (Fig. 5D).

### Validation of six differentially-expressed secreted protein genes

In order to verify the reliability of RNA-seq results, we analyzed the expression levels of six shared but differentially-expressed genes (ecdysone oxidase, yellow-d, cell wall protein IFF6, lipase 3, serine protease inhibitor dipetalogastin, aldehyde dehydrogenase) between *H. armigera* and *H. assulta* by RT-qPCR. RNA-seq showed that the RPKM values of the six genes in the labial salivary glands were 11 to 627-fold greater in *H. armigera* than in *H. assulta* (Table 8). Consistent with the RNA-seq results, RT-qPCR showed that the normalized transcript levels of the six genes were 2.4-317.7-fold greater in *H. armigera* than in *H. assulta* (Table 8), indicating that our sequencing data are reliable.

**Table 8:** RT-qPCR verification of transcripts level of candidate genes in salivary glands of *H. armigera* and *H. assulta*

Gene	Means of normalized transcripts level ± standard errors		*H. armigera* / *H. assulta* ratio of transcripts level	*P*-value (Difference level of *H. armigera* and *H. assulta*)	RPKM		*H. armigera* / *H. assulta* ratio of RPKM
	*H. armigera*	*H. assulta*			*H. armigera*	*H. assulta*	
Ecdysone oxidase (EO)	3.29 ± 0.15	0.68 ± 0.02	4.84	0.005	2728.85	4.35	627.041
Yellow-d (YD)	2.63 ± 1.05	1.09 ± 0.29	2.41	0.230	7839.53	675.38	11.61
cell wall protein IFF6 (CWP)	3296.14 ± 37.89	12.70 ± 5.00	259.61	0.002	4729.45	314.55	15.04
lipase 3 (LP-3)	6.24 ± 0.21	1.74 ± 0.20	3.57	0.000	12540.68	1132.44	11.07
serine protease inhibitor dipetalogastin (SPI_D)	91.72 ± 11.76	0.29 ± 0.18	317.66	0.016	10174.66	18.14	560.76
aldehyde dehydrogenase (AD)	0.78 ± 0.14	0.12 ± 0.02	6.53	0.011	1050.44	44.93	23.38

## Discussion

The recent finding of an inverse relationship between the genome size and host plant range in the *Helicoverpa* lineage [1] implies that the generalist *H. armigera* should have a larger secretome size than its closely-related Solanaceae specialist *H. assulta.* This may be manifested by the inter-species difference in the transcriptome size [i.e. the total numbers of contigs (or transcripts) and unigenes] of the labial salivary gland, a major tissue for secretion of effectors-containing (e.g., plant defense-inhibiting glucose oxidase, ATP hydrolyzing enzymes) saliva to manipulate/modify host plant nutrients, structure and defense [15–24]. Consistent with this prediction, the numbers of total contigs, total unigenes and species-specific contigs expressed in the labial salivary gland of *H. armigera* were 1.88 (85801 / 45547), 1.91 (69340 / 36345) and 2.33 (70493 / 30239) times more than those in the labial salivary gland of *H. assulta* (Tables 1and Fig. 1), respectively. By contrast, the *H. armigera*/*H. assulta* ratios of the number of total contigs in the host-adapting midgut and fat body were 1.20 and 1.15 respectively (unpublished data). Moreover, the numbers of total contigs and unigenes expressed in the host plant-locating antennae [26–28] were 1.19 (97631 / 82205) and 1.05 (53479 mix / 50763 female)-1.21 (53479 mix / merged 44319) times more in *H. armigera* than in *H. assulta* [50], respectively. Along the same line, the *H. armigera* / *H. assulta* ratios were 1.25 (44096 / 35356) for the total number transcripts (i.e. contigs) in the sex pheromone gland [48], an insect tissue unrelated to host plant use, and 0.996 (53435 / 53268) for the total number of unigenes in the sex pheromone gland-ovipositor complex [49]. Similarly, whole body transcriptome comparison revealed a *H. armigera* / *H. assulta* ratio of 0.998 (356842 / 357414) for the total number of transcripts expressed in four developmental stages (egg, larvae, pupae, adults), 1.06 (70012 / 66038) for the species-specific transcripts in eggs, 0.88 (53183/60681) for the species-specific transcripts in larvae, 1.01 (60440 / 59958) for the species-specific transcripts in pupae, and 1.04 (61736 / 59266) for the species-specific transcripts in adults [69].

Notably, the larger size of the labial salivary gland transcriptome of *H. armigera* is not just limited to the secretome (secreted proteins) that directly participates in the modification of host plants. This is evidenced by several findings from this study. First, the numbers of species-specific contigs of the labial salivary gland transcriptome in all level 2 GO functionary categories (Fig. 2B) were greater in *H. armigera* than in *H. assulta*. This is in sharp contrast to the transcriptomes of the sex pheromone gland [48] and antennae [50], whereby no such broad *H. armigera* biases were found. Second, we detected significant enrichment and/or more contigs for all the selected GO terms and KEGG pathways (except for vitamin digestion and absorption (ko04977)] associated with the major functions of the labial salivary gland rather than just those [e.g. oxidoreductase (GO:0016491), hydrolase (GO:0016817), exopeptidase (GO:0008238), Drug metabolism - other enzymes (ko00983) and nicotinate and nicotinamide metabolism (ko00760)] related to herbivore offense, detoxification, digestion and/or immunity [70] (Tables 2 and 3). The *H. armigera* / *H. assulta* size ratios were 4.4 (hydrolase activity) to 32.4 (aminoacyl-tRNA ligase activity) for salivary gland-related molecular function terms, 4.9 (protein metabolic process) to 32.4 (tRNA aminoacylation for protein translation) for salivary gland-related biological process terms, and 4.8 (Golgi apparatus part) to 52.5 (aminoacyl-tRNA synthetase multienzyme complex) for salivary gland-related cellular component terms (Table 2). In fact, the GO terms or pathways with the highest *H. armigera* / *H. assulta* size ratios were for protein synthesis / secretion, such as protein export pathway (70.75), tRNA aminoacylation for protein translation (32.4) and aminoacyl-tRNA synthetase multienzyme (52.5), rather than for herbivore offense, detoxification and digestion such as oxidoreductase (7.1), hydrolase (4.4), exopeptidase (4.8) and detoxification (30) (Tables 2 and 3). Even for the top 30 enriched GO terms and top 20 enriched KEGG pathways of *H. assulta*, there were still 23 GO terms (Table 5) and 18 pathways (Table 7) with a *H. armigera* / *H. assulta* size ratio of greater than 1.

The larger size of the labial salivary transcriptome of *H. armigera* is also manifested at the gene level. As an evidence, *H. armigera* expressed 1.88 times more species-specific genes than *H. assulta* in the labial salivary gland (Fig. 4A). The magnitude of their difference decreased to 1.5-fold for the species-specific essential genes but increased to 2.52-fold for the species-specific environmental response genes (Fig. 5A). The numbers of the total and species-specific putative secreted protein genes bioinformatically identified were 1.45 and 1.85 times more in *H. armigera* than in *H. assulta* (Fig. 6A), respectively. By contrast, the ratios for *H. armigera* versus *H. assulta* were 1.16 (108/93) for the total number of putative pheromone biosynthesis, transport and degradation genes expressed in the sex pheromone gland [48], 1.05 (90/86) for the total number of pheromone production related genes expressed in the sex pheromone gland-ovipositor complex [49], and 1.02 (133/131) for the total number of putative chemosensory genes expressed in the antennae [50].

While not necessarily implied by the inverse relationship between host range and genome size in the *Helicoverpa* lineage [1], the findings of the current study showed that *H. armigera* tended more alleles per gene (Figs. 4B and 5B and B) and higher gene expression level than did *H. assulta* (Figs. 3C, 4C and 5C, and Table 8). These differences may allow *H. armigera* to overcome the diversity of nutrient / defense challenges encountered in its broad range of host plants when compared to the specialist *H. assulta*. The finding that the generalist *H. armigera* had more alleles per gene (i.e. greater allelic diversity within a locus) than its sister specialist *H. assulta* agrees with the specialist-generalist variation hypothesis (SGVH), which predicts that generalists should have higher genetic diversity but less population differentiation [71]. Previously, Kelley et al. (2000) [72] found that the generalist bark beetle *Dendroctonus ponderosae* kept approximately 10 times the genetic variation in mitochondrial DNA as its sister specialist *Dendroctonus jeffrey.* Additionally, Zayed et al. (2005) [73] showed that the generalist bee *Colletes seminitidus* maintained about 5 times the heterozygosity at 15 allozyme loci as its closely-related specialist bee *Leioproctus rufiventris*. Clearly, our work and the above two previous studies prove the validity of the SGVH in insect herbivores.

That *H. armigera* has a larger transcriptome than *H. assulta* in the host modification tissue labial salivary gland (this study) but not in the host-location tissue antennae [50] and host use-unrelated tissues sex pheromone gland [48] and its complex with ovipositor [49] supports the notion that the generalist *Helicoverpa* species rely more on host modification than host adaptation to achieve polyphagy [1]. A further question that arises is why the generalist *Helicoverpa* species have more host use-related genes expressed in the host-modifying salivary gland, and probably also in the host-adapting midgut and fat body than their specialist siblings. One possibility is that those extra host use genes expressed in the host modification and adaptation tissues of the generalist *Helicoverpa* species are absent in the genomes of the specialist siblings because they are no longer needed due to narrow diet breadth. Alternatively, most of those unneeded genes, if not all, still exist but are pseudogenized or turned off due to increased TE activation/proliferations (thus larger genome) and transposition frequency, as we recently speculated [1]. In agreement with the latter hypothesis, among the top 30 enriched GO terms of the *H. assulta* labial salivary gland transcriptome, 5 GO terms were associated with TE transposition, but these terms were not enriched in the *H. armigera* labial salivary gland transcriptome, and their *H. armigera* / *H. assulta* size ratios were < 1 (Tables 4 and 5). In line with this finding, the percentage of contigs containing TE was significantly higher in the *H. assulta* labial salivary gland transcriptome than in the *H. armigera* labial salivary gland transcriptome (Fig. 3A and C). Moreover, the genomic content of TEs was significantly higher in *H. assulta* (33.01%) [67] than in *H. armigera* (11.33%) [66] (Fig. 3D). Even more convincingly, *H. assulta* genome possess a higher proportion of pseudogenes in the P450, UGT, CCE and GST families than *H. armigera* genome, while the two species do not differ significantly in the total number of genes in the four detoxification families (Fig. 3E and H) [67, 68]. Taken together, these results confirm that oscillation of host plant range in the *Helicoverpa* lineage is accompanied by TE-mediated pseudogenization (shutdown) and reactivation (on) of host use and secretion related genes. This represents to our knowledge the first report of the mechanisms that facilitate oscillation of host-plant range evolution in herbivorous insects.

Recent comparative studies on the generalist *Spodoptera frugiperda* and its specialist relative *Spodoptera picta* suggest that TE-mediated pseudogenization (shutdown) and reactivation (on) of host use related genes may also facilitate oscillation of host plant range in the *Spodoptera* lineage, which, like the *Helicoverpa* genus, also belongs to the Noctuidae family of Lepidoptera. The first supporting evidence is that the genome size of the specialist *S. picta* (486 Mb) [74] is significantly larger than that of its within-genus generalist *S. frugiperda* (380-390 Mb) [75–77]. Another attestation is the significant pseudogenization of UGT33 and UGT40 families, two UGT gene families known to enable *S. friugiperda* to utilize the poaceous plants (such as maize, wheat, and rice) and cotton, respectively, occurred only in the specialist *S. picta*, but not in *S. frugiperda* [74]. Further comparative studies are needed on TE content and pseudogenization of host utilization related genes in more lineages to determine whether TE-mediated pseudogenization and reactivation of host use related genes is a common or lineage-specific mechanism for oscillations in host-plant range of herbivorous insects.

## Supplementary Information

Supplementary Material 1.

Supplementary Material 2.

## Data Availability

The reads data of *H. armigera* and *H. assulta* labial salivary glands were de novo assembled into contigs according to Sect.  4.4. The assembled transcriptomes of *H. armigera* and *H. assulta* have been deposited in European Nucleotide Archive (ENA) under Study ID PRJEB43360, Sample ID ERS5843106 for *H. armigera* and ERS5843107 for *H. assulta* , Sequence accession HBTK01000001-HBTK01085801 for *H. armigera* and HBTJ01000001-HBTJ01045547 for *H. assulta* .
