# Supplementary material for: Transcriptomic divergences of larval labial salivary glands facilitate host-plant range oscillations between specialist and generalist Helicoverpa species
Source: BMC Genomics. 2026 Apr 2;27:465. doi: 10.1186/s12864-026-12774-z (PMC13170022; doi:10.1186/s12864-026-12774-z)
Supplement: Supplementary file 1 — Supplementary Material 1. [file 12864_2026_12774_MOESM1_ESM.docx]

**Table S1. Primer pairs used for qRT-PCR verification.**

| **Gene** | **Primer sequences (5′-3′)** | **Length of product (bp)** | **Primer amplification efficiency (%)** | |  | **R^2^** | |
| --- | --- | --- | --- | --- | --- | --- | --- |
|  |  |  | ***H. armigera*** | ***H. assulta*** |  | ***H. armigera*** | ***H. assulta*** |
| ecdysone oxidase (EO2) | F: GTCAGCGTGCTACTGCTAGAA | 169 | 0.9470 | 0.9097 |  | 0.9964 | 0.9906 |
|  | R: GCCTTGAGTCAGACCGACATGG |  |  |  |  |  |  |
| yellow-d (YD) | F: GCGACCGACCAGCTCATCTACA | 167 | 0.9369 | 0.9593 |  | 0.9985 | 0.9991 |
|  | R: TCCACCACGAGGATTGCGAAGG |  |  |  |  |  |  |
| cell wall protein IFF6 (CWP) | F: ACTGGCAACAACAACGGATGGT | 93 | 0.9615 | 0.9785 |  | 0.9983 | 0.9999 |
|  | R: TGCTACCGCTGAACCGACCT |  |  |  |  |  |  |
| lipase 3 (LP-3) | F: TTGCCTGTGGTCGTTGGTCAC | 151 | 0.9391 | 0.9239 |  | 0.9990 | 0.9994 |
|  | R: CGTACAGTGGCGGACTCCTCTT |  |  |  |  |  |  |
| serine protease inhibitor dipetalogastin (SPI_D) | F: GCTGAGGTGCGAGAACAAGAGG | 156 | 0.9301 | 0.9273 |  | 0.9993 | 0.9999 |
|  | R: AGCCAGCAGCGGTTCCAGTA |  |  |  |  |  |  |
| aldehyde dehydrogenase (AD) | F: GTCGCCTCCTCCTCAAGTTAGC | 132 | 0.9011 | 0.8725 |  | 0.9987 | 0.9991 |
|  | R: TGATGATGCCAGCAGACCACAC |  |  |  |  |  |  |
| ribosomal protein L-13 (RPL-13) | F: CTGCAAGACGTCACCGCAG | 139 | 1.003 | 0.9301 |  | 0.9981 | 0.9999 |
|  | R: CCACGACCAGCACGAACCT |  |  |  |  |  |  |
| ribosomal protein L-32 (RPL-32) | F: CATCAATCGGATCGCTATG | 152 | 1.0043 | 0.9349 |  | 0.9990 | 0.9954 |
|  | R: CCATTGGGTAGCATGTGAC |  |  |  |  |  |  |
| β-Tubulin (β-TUB) | F: AGCAGTTCACCGCTATGTTC | 106 | 1.0834 | 0.8997 |  | 0.9997 | 0.9995 |
|  | R: AGGTCGTTCATGTTGCTCTC |  |  |  |  |  |  |
